# Molecular Science of Eicosapentaenoic Acid (EPA) Omega-3 Fatty Acid Using *Nannochloropsis oceanica* as a Model Organism

**DOI:** 10.3390/ijms27219834

**Published:** 2026-11-02

**Authors:** Evan D. Stephens, Skye Thomas-Hall, Victor Joseph Galea, Kirsten Heimann

**Affiliations:** 1 School of Agriculture and Food Sustainability, University of Queensland, St Lucia, QLD 4072, Australia; v.galea@uq.edu.au; 2 Algemy Pty Ltd., Brisbane, QLD 4035, Australia; 3 Advantage Plants Consulting Pty Ltd., Brisbane, QLD 4500, Australia; 4 SeoO2 Pty Ltd.,Rozelle, NSW 2039, Australia; skyethomashall@gmail.com; 5 Faculty of Science and Engineering, Southern Cross University, Military Road, P.O. Box 157, East Lismore, NSW 2480, Australia

**Keywords:** eicosapentaenoic acid, EPA, omega-3 fatty acids, *Nannochloropsis oceanica*, marine microalgae, biosynthesis, lipid metabolism, specialized pro-resolving mediators, resolvins, arachidonic acid to eicosapentaenoic acid ratio, inflammation, cardiovascular disease

## Abstract

The EPA-rich marine microalga *Nannochloropsis oceanica* provides insight into EPA biosynthesis via the characterized Δ6-desaturase pathway, positioning this microalga for both experimental research and sustainable production solutions for non-fish oil-derived EPA oils. This review constructs a comprehensive molecular-to-clinical framework demonstrating how the chemical structure of eicosapentaenoic acid (EPA) determines its biological activities, i.e., the specific positioning of the Δ17 double bond governs competitive enzymatic metabolism with arachidonic acid (ARA), yielding attenuated 3-series and 5-series eicosanoids while uniquely enabling E-series resolvin biosynthesis. Metabolic pathway modulation (AMPK, NF-κB, and SREBP-1c) translate biochemical events into transcriptional reprogramming. Sustained alterations in gene expression, membrane composition, and inflammatory mediator profiles manifest as clinical benefits across cardiovascular disease, metabolic disorders, inflammatory conditions, and psychiatric illnesses. The pleiotropic nature of EPA effectssimultaneously anti-inflammatory, TAG-lowering, anti-arrhythmic, insulin-sensitizing, and pro-resolving — arises not from independent mechanisms but from coordinated, multi-level integration. The ARA:EPA ratio emerges as the unifying quantitative biomarker, integrating dietary intake, competitive membrane incorporation, eicosanoid biosynthetic balance, and tissue-level inflammatory tone into a single metric predictive of disease risk and therapeutic response. Lowering this ratio through EPA supplementation represents a systems-level intervention: simultaneously reducing pro-inflammatory mediator production, enhancing resolution capacity, improving metabolic flexibility, and stabilizing cellular energetics.

## 1. Introduction

Eicosapentaenoic acid (EPA, 20:5n-3) plays a pivotal role in omega-3 polyunsaturated fatty acid (ω-3 PUFA) biology, distinguished by its 20-carbon backbone and five methylene-interrupted cis double bonds, which is outlined by the NCBI’s PubChem resource along with the other essential fatty acids discussed in this study [1]. First, isolated from fish liver oils in the 1950s and structurally characterized in the 1960s, EPA was initially recognized as a quantitatively abundant component of marine lipids. The systematic nomenclature—all-cis-5,8,11,14,17-eicosapentaenoic acid—precisely defines the positions of unsaturation when numbered from the carboxyl terminus, while the shorthand designation 20:5n-3 indicates 20 carbons and five double bonds, with the terminal double bond located at the third carbon from the methyl end, classifying it as an omega-3 fatty acid (ω-3 FA) [1].

The biological significance of EPA emerged progressively through landmark epidemiological observations in Greenland Inuit populations during the 1970s, revealing inverse associations between high marine ω-3 PUFA consumption and cardiovascular disease prevalence [2]. Subsequent mechanistic investigations established EPA as both a structural membrane component and a precursor to bioactive lipid mediators—3-series prostaglandins, 5-series leukotrienes, and E-series resolvins—that modulate inflammation, thrombosis, and tissue resolution processes [3]. Unlike its plant-derived precursor α-linolenic acid (ALA, 18:3n-3) [1], which exhibits minimal bioconversion to EPA efficiency in humans (~5–10% to EPA, <1% to docosahexaenoic acid (DHA, C22:6n-3)) [4], preformed EPA from marine sources directly influences tissue phospholipid composition and eicosanoid biosynthesis. Thus, while EPA is theoretically synthesizable from ALA, low physiological conversion rates necessitate direct dietary intake to achieve tissue concentrations associated with clinical benefits, making it an essential fatty acid (EFA).

Contemporary interest in EPA has intensified following large-scale clinical trials demonstrating cardiovascular event reduction with high-dose purified EPA formulations (REDUCE-IT [5] and JELIS [6]), mechanistic elucidation of specialized pro-resolving mediator (SPM) pathways unique to EPA [7], and growing recognition of the ARA [1]-to-EPA ratio as a quantitative biomarker for inflammatory status, metabolic health, and disease risk [8]. Concurrently, biotechnological advances in marine microalgae cultivation—particularly *Nannochloropsis oceanica* (see Supplementary Materials S1)—have enabled sustainable, scalable production of EPA-rich oils [9], offering alternatives to traditional fish-derived sources while providing controlled model systems for investigating EPA biosynthesis and metabolism.

### Scope and Rationale

Despite EPA’s established clinical importance and expanding therapeutic applications, existing reviews typically focus on either molecular mechanisms or clinical outcomes divorced from mechanistic context. This fragmentation obscures the essential continuum linking EPA’s chemical structure to its diverse physiological effects. Comprehensive, molecularly informed knowledge synthesis through integrating chemical properties, biosynthetic pathways, enzymatic transformations, pharmacokinetics, genomic regulation, and clinical translation is notably absent from the literature.

This review addresses this gap by constructing an integrated molecular-to-clinical framework centered on EPA, using *N. oceanica* as a model organism to illuminate biosynthetic mechanisms while maintaining clinical relevance.

The review integrates across traditional disciplinary boundaries: physical organic chemistry (structure–function relationships governing EPA’s reactivity and membrane behavior), enzymology (competitive substrate metabolism with ARA and specialized pro-resolving mediator biosynthesis), molecular biology (gene regulation via nuclear receptors, transcription factors, and epigenetic modifications), pharmacology (absorption, distribution, metabolism, and elimination kinetics), and clinical medicine (cardiovascular protection, anti-inflammatory effects, and metabolic benefits). This integration reveals emergent properties—such as the ARA:EPA ratio as a unifying biomarker for disease risk or prevalence—that are invisible within single-discipline perspectives.

This consolidated information and molecular insights are relevant to research on lipid biochemistry, inflammation, and metabolic regulation; biotechnology approaches to algal production systems; clinical investigations designing ω-3 intervention trials; and industry formulating EPA-based nutritional and pharmaceutical products. By maintaining molecular rigor while explicating clinical relevance, this review serves both specialist and translational readership.

## 2. Chemical Structure, Primary ω-3 EFA Properties, and Sources

**Figure 1:**
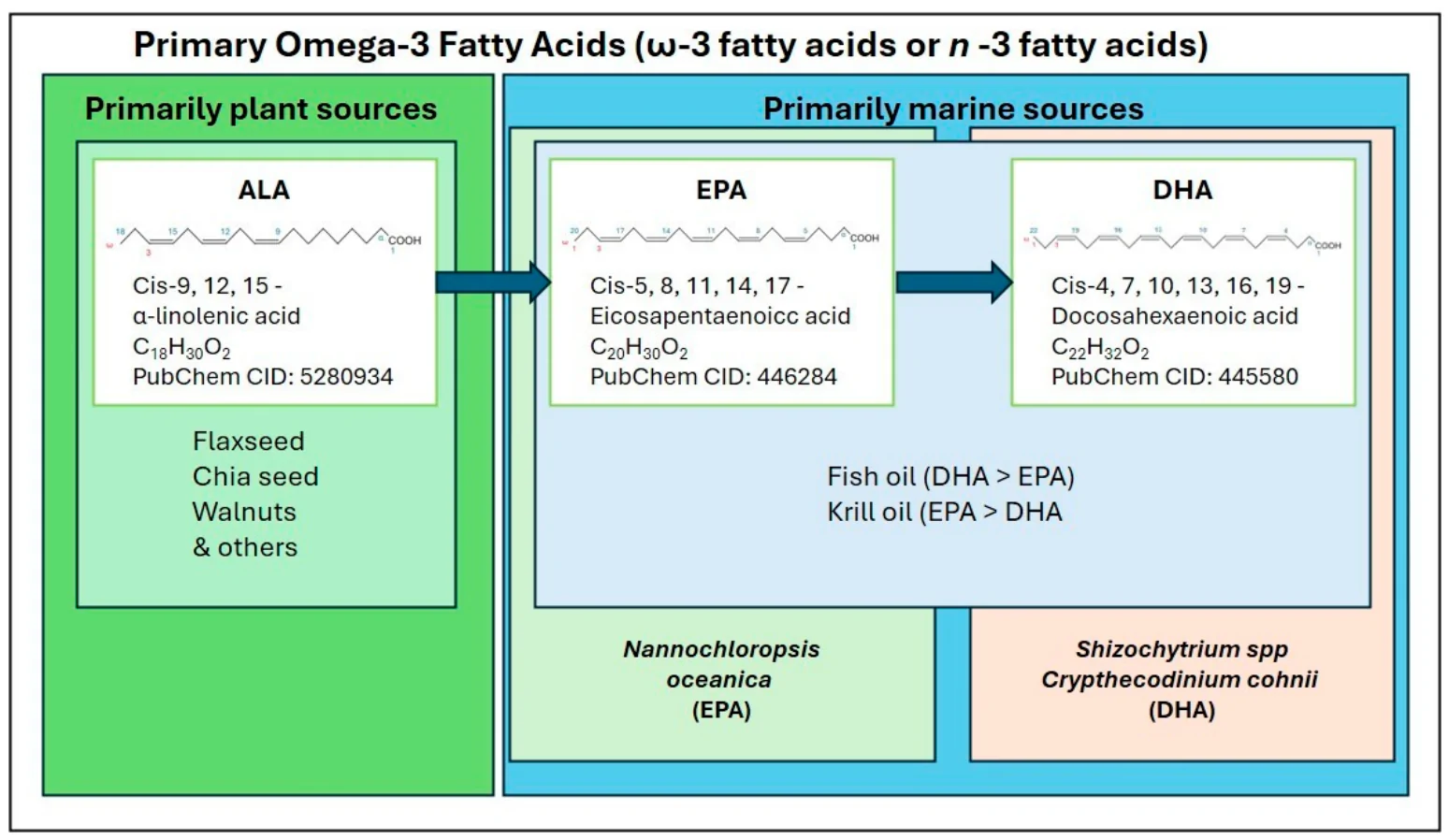
Molecular structures of primary ω-3 EFAs, biosynthetic relationships, and natural sources. The three principal dietary ω-3 PUFAs are distinguished by chain length and the degree of unsaturation. ALA (18:3n-3) is derived predominantly from plant sources including flaxseed, chia seeds, and walnuts. ALA serves as the metabolic precursor for longer-chain ω-3 EFAs. EPA and DHA are obtained from marine sources including fish oil (typically DHA > EPA) and krill oil (typically EPA > DHA), whose content of these EFAs is generally acquired through direct or indirect dietary intake of microscopic phytoplankton and zooplankton, e.g., marine microalgae *Nannochloropsis oceanica,* thraustochytrids *Schizochytrium* sp., and the dinoflagellate *Crypthecodinium cohnii*, although there are numerous other types in nature. Molecular structures are shown in the skeletal formula with systematic IUPAC nomenclature. Arrows indicate biosynthetic pathway progression.

**Figure 2:**
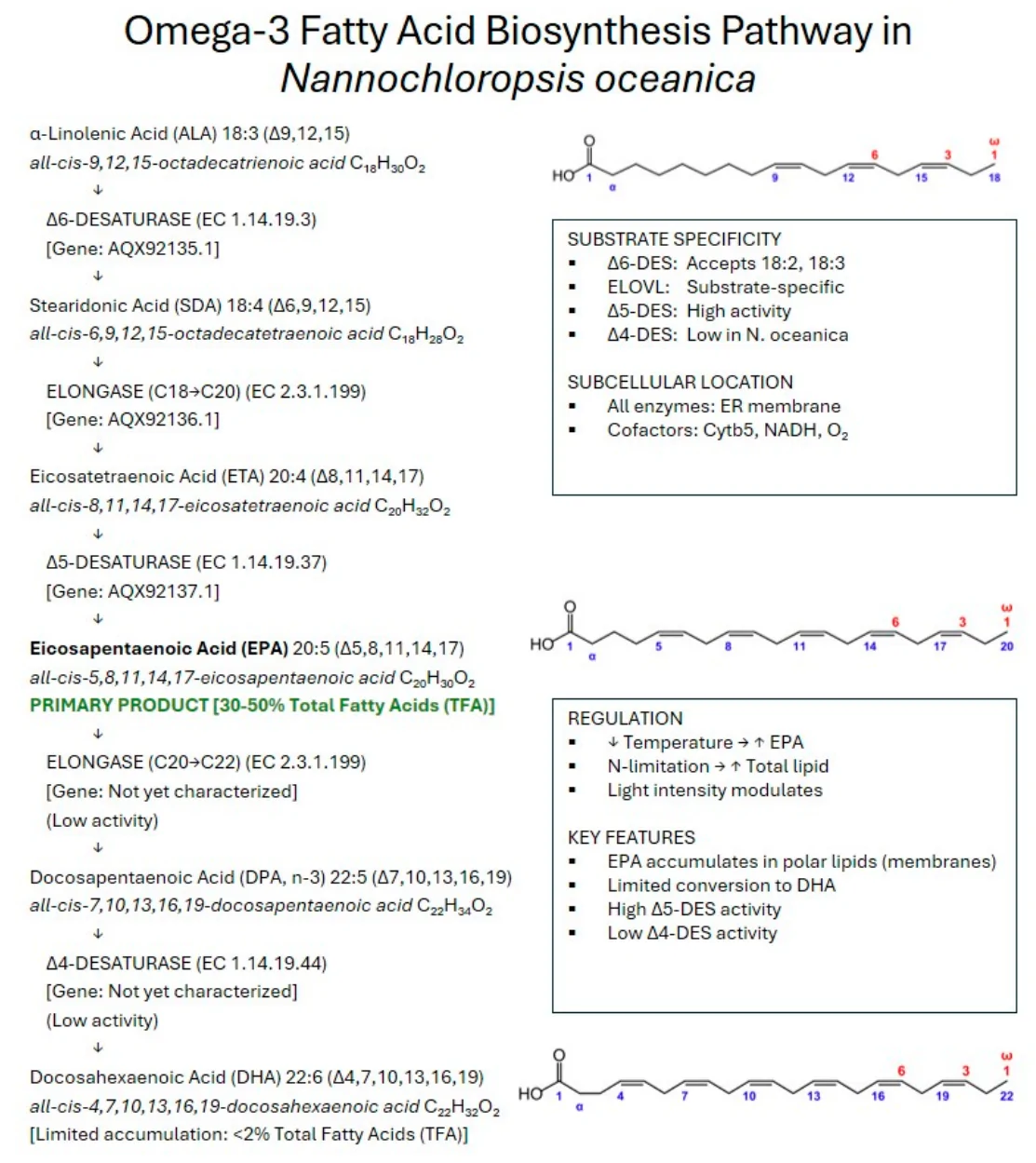
Characterized EPA biosynthesis pathway in *Nannochloropsis oceanica* CCMP531/1779. *Nannochloropsis oceanica* synthesizes EPA via a well-characterized Δ6-DES pathway. ALA (18:3Δ9,12,15) undergoes Δ6-desaturation (GenBank: AQX92135.1) to stearidonic acid (SDA, 18:4Δ6,9,12,15), which is elongated by Δ6-elongase (GenBank: AQX92136.1) to eicosatetraenoic acid (ETA, 20:4Δ8,11,14,17). Δ5-desaturation (Δ5-DES, GenBank: AQX92137.1) yields EPA (20:5Δ5,8,11,14,17). Elongation of EPA to docosapentaenoic acid (DPA, 22:5Δ7,10,13,16,19) and subsequent Δ4-desaturation to DHA (22:6Δ4,7,10,13,16,19) proceed with minimal flux, resulting in negligible DHA accumulation (<2% TFA). Genes encoding C20→C22 elongase and Δ4-DES have not been characterized in *N. oceanica* CCMP531/1779, consistent with the EPA-rich phenotype. This contrasts with DHA-producing microorganisms such as dinoflagellates, thraustochytrids, and prymnesiophytes, which possess highly active Δ4-DES. All characterized enzymes are ER membrane-bound and require cytochrome b5, NADH, and O_2_. Pathway flux is regulated by temperature (inverse correlation with EPA %), nitrogen availability, and light intensity. EC numbers indicate enzyme classification; GenBank accessions are for *N. oceanica* CCMP531/1779. TFA = total fatty acids; ER = endoplasmic reticulum.

**Figure 3:**
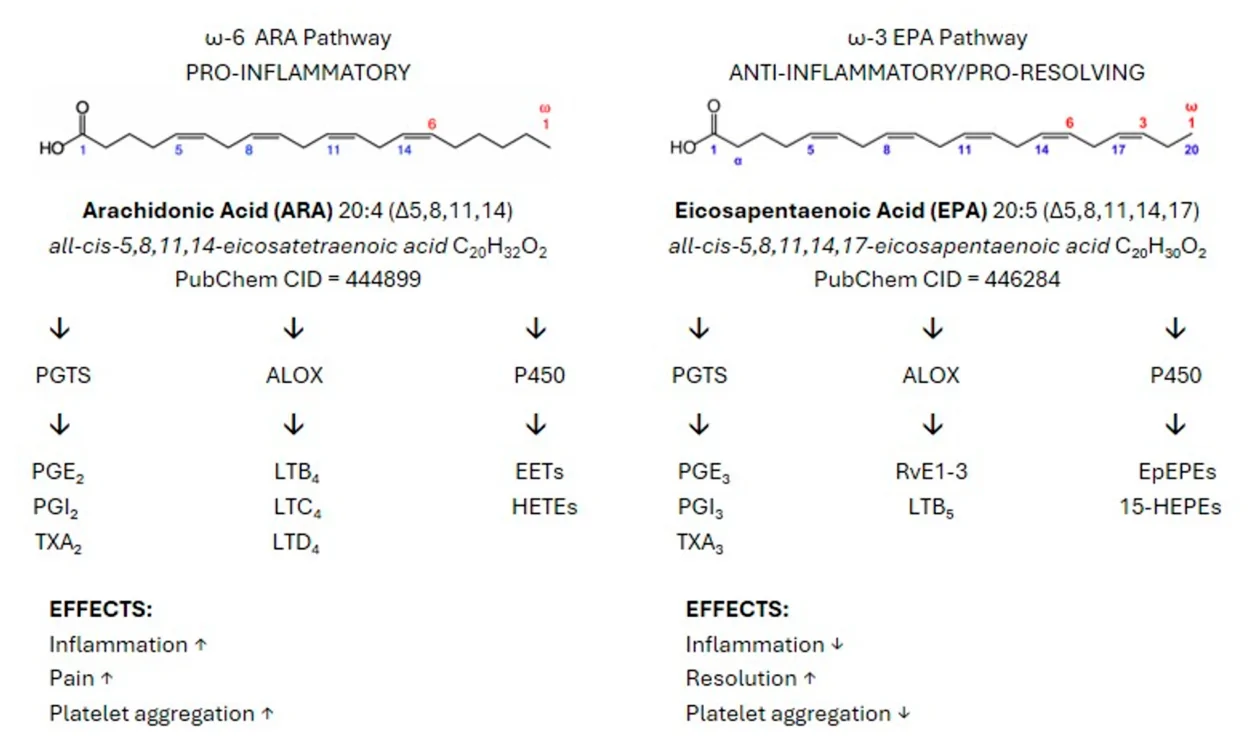
ω-6 ARA and ω-3 EPA roles in inflammation balance and processes and competing eicosanoid pathways. ARA:EPA ratio as biomarker: The ratio of ARA to EPA in plasma or red blood cell membranes is increasingly recognized as a quantitative health biomarker. They are metabolized to distinctly different immunomodulatory compounds, resulting in different influences on inflammation. PTGS = prostaglandin synthesase; ALOX = lipoxygenase; P450 = cytochrome P450; PG = prostaglandin; TX = thromboxane; LT = leukotriene; EET = epoxy-eicosatrienoic acid; HETE = hydroxy-eicosatetraenoic acid; EpEPE = epoxy-eicosapentaenoic acid; HEPE = hydroxy-eicosapentaenoic acid; RvE = resolvin (E-series).

Understanding the unique properties of EPA requires comparative analysis with structurally related ω-3 EFAs—ALA and DHA—and the ω-6 counterpart ARA. This section integrates previous discussions (Figure 1, Figure 2 and Figure 3; Table 1 and Table 2) to position EPA within the broader landscape of PUFA biology and clinical nutrition [10].

The systematic IUPAC nomenclature of EPA is all-cis-5,8,11,14,17-eicosapentaenoic acid, precisely defining the positions of unsaturation when numbered from the carboxyl terminus. Alternative shorthand designations include 20:5n-3 (indicating 20 carbons and five double bonds, with the first double bond at the third carbon from the methyl terminus) or 20:5ω-3 using Greek letter notation. The molecular formula for EPA is C_20_H_30_O_2_ (Figure 1), resulting in a molecular weight of 302.45 g/mol (PubChem CID: 446284) [1]. The five cis-configured double bonds at Δ5, Δ8, Δ11, Δ14, and Δ17 positions impose significant conformational constraints, each introducing a ~30° bend in the carbon chain, resulting in a highly kinked, three-dimensional structure (Figure 1) that contrasts sharply with the extended conformation of saturated FAs. This geometric configuration fundamentally determines the unique physicochemical properties and biological activities of EPA. The separation of each double bond by a single methylene (-CH_2_-) group is characteristic for mammalian and algal PUFAs and distinguishes EPA from dietary-derived conjugated polyunsaturated systems found in some plant species. In comparison to EPA, the carbon chain of the plant-derived ω-3 precursor ALA (18:3n-3) [1] is two carbon atoms shorter and possesses only three double bonds, while the carbon chain of DHA (C22:6n-3) [1] is two carbon atoms longer with an additional double bond (Figure 1). Relative to eicosatetraenoic acid (20:4n-3, the immediate biosynthetic precursor), EPA possesses an additional double bond at Δ5 [11]. Critically, EPA differs from ARA (20:4n-6) by a single double bond at position Δ17 [1]; this seemingly minor structural variation profoundly alters the spectrum of eicosanoid metabolites produced and their biological potencies (Figure 3) [11].

The EPA biosynthetic capacity of *N. oceanica* can be contextualized against other natural sources (Figure 1). Marine fish accumulate EPA primarily through dietary intake from microalgae and zooplankton, with limited endogenous synthesis; fish oil EPA content varies by species, diet, and environmental conditions but typically ranges from 5–20% of total FAs, usually accompanied by comparable or higher DHA levels (Figure 1). Krill (*Euphausia superba*) oil exhibits a more EPA-dominant profile (EPA:DHA ratios often >1:1) but still contains significant amounts of DHA (Figure 1) [12].

In contrast, *N. oceanica* produces EPA-dominant oils (30–50% EPA, <2% DHA) with a fatty acid profile optimized for EPA delivery without substantial DHA [9,13]. This positions *N. oceanica*-derived oils as distinct from the more traditional marine sources, offering a controlled, sustainable, and EPA-selective alternative [14]. Other *Nannochloropsis* species (*N. gaditana*, *N. salina*, and *N. granulata*) exhibit similar EPA-rich phenotypes, though strain-specific variations in EPA content and lipid productivity exist [15] (see Supplementary Information).

Higher plants generally lack Δ6- and Δ5-desaturases and therefore cannot synthesize EPA from ALA (Figure 1, Table 1). Humans possess these desaturases but exhibit low conversion efficiency (ALA→EPA conversion ~5–10%) [4], necessitating direct EPA intake for achieving therapeutic tissue levels. Genetically engineered plants expressing algal or fungal desaturase/elongase genes have been developed experimentally to produce EPA, but *N. oceanica* and related microalgae remain the predominant biotechnological platform for large-scale EPA production due to their naturally high biosynthetic capacity, rapid growth, and established cultivation infrastructure [10].

## 3. Physicochemical Properties of EPA

The high degree of unsaturation confers distinctive physicochemical characteristics that influence EPA’s behavior in biological systems. The melting point of pure EPA is ~54 °C, substantially lower than for the saturated arachidic acid (20:0; mp 75 °C) or mono-unsaturated eicosenoic acid (20:1; mp ~25 °C) (see Supplementary Information). The low melting point results from the inability of kinked cis double bonds to pack efficiently into crystalline arrays, maintaining EPA in a fluid state at physiological temperatures. Consequently, EPA-enriched membrane phospholipids exhibit enhanced fluidity, facilitating protein conformational dynamics, receptor–ligand interactions, and lateral lipid domain organization. Like other long-chain fatty acids, EPA is essentially insoluble in water (estimated aqueous solubility < 0.1 mg/L at 25 °C) but highly soluble in non-polar organic solvents including chloroform, hexane, and ethanol. This pronounced lipophilicity, quantified by an octanol–water partition coefficient (log P) of ~6.8 [16], dictates EPA’s partitioning into hydrophobic cellular compartments—primarily membrane bilayers and lipid droplets—and necessitates protein-mediated transport in aqueous biological fluids. In plasma, EPA is transported predominantly within lipoprotein particles (chylomicrons, VLDL, LDL, and HDL) and bound to albumin; unesterified EPA concentrations are tightly regulated in the low micromolar range [17]. The multiple cis double bonds render EPA highly susceptible to oxidative degradation [18]. Bis-allylic hydrogen atoms—those positioned between two double bonds (at C-7, C-10, C-13, and C-16)—exhibit bond dissociation energies of ~15 kcal/mol lower than allylic or vinylic hydrogens, making them preferential sites for free radical abstraction [19]. Autoxidation proceeds via formation of conjugated diene hydroperoxides, which can further decompose to aldehydes, ketones, and epoxides [19]. This oxidative lability necessitates careful handling during extraction, storage, and formulation; antioxidants such as tocopherols are routinely employed in EPA-containing products to minimize peroxidation. Paradoxically, controlled enzymatic oxidation of EPA by cyclooxygenases, lipoxygenases, and cytochrome P450 enzymes generates bioactive signaling molecules central to EPA’s physiological functions [3,11]. Spectroscopic properties enable EPA detection and quantification. The conjugated diene system formed upon oxidation yields a characteristic absorption at 234 nm, providing a sensitive marker of lipid peroxidation, while the absorption peak of unoxidized EPA is 200–210 nm [20]. Nuclear magnetic resonance (^1^H-NMR and ^13^C-NMR) spectroscopy provides definitive structural characterization: ^1^H-NMR signals for olefinic protons appear at δ 5.2–5.4 ppm, bis-allylic methylene protons at δ 2.7–2.8 ppm, and the terminal methyl group at δ 0.9 ppm. Gas chromatography–mass spectrometry (GC-MS) of EPA methyl esters yields diagnostic fragmentation patterns, with the molecular ion at *m*/*z* 316 and characteristic fragments enabling unambiguous identification in complex lipid extracts [21].

## 4. Biosynthesis and Metabolism of EPA

### 4.1. The Δ6- and Δ4-Desaturase Biosynthetic Pathway for Microalgal EPA and DHA Production

The genus *Nannochloropsis* (Eustigmatophyceae) belongs to the monophyletic group Stramenopiles, who are, except for the heterotrophic thraustochytrids, characterized by high contents of EPA and low contents of DHA [9]. In these organisms, accumulation of DHA is limited due to the low or absent activity of enzymes involved in the terminal steps of C20→C22 elongation, as shown for *N. oceanica* CCMP531/1779. Specifically, the genes encoding C20→C22 elongase and Δ4-desaturase (Δ4-DES; EC 1.14.19.44) have not been characterized in this strain (Table 1, Figure 2) [9]. GenBank searches yield no characterized sequences for these enzymes, and FA profile analyses consistently show DHA levels below 2% of total FAs (Figure 2) [9,14]. This metabolic bottleneck—whether due to gene downregulation, extremely low expression, post-transcriptional repression, or enzyme instability—results in EPA serving as the metabolic endpoint rather than an intermediate enroute to DHA. This EPA-dominant phenotype distinguishes *N. oceanica* from DHA-producing microorganisms, such as dinoflagellates and thraustochytrids (Figure 1), which possess highly active Δ4-DES enabling efficient DHA synthesis [22]. The molecular basis for this difference—high Δ5-desaturase (Δ5-DES) activity coupled with low/absent Δ4-DES activity—positions *N. oceanica* as an ideal biotechnological platform for producing EPA-rich oils without substantial DHA contamination [9]. For applications requiring pure or EPA ω-3 profiles, this metabolic configuration is advantageous.

Like in other EPA-producing microalgae, EPA is synthesized in *N. oceanica* via the crucial Δ6-desaturase (Δ6-DES) pathway, a conserved eukaryotic route for long-chain ω-3 PUFA biosynthesis (Figure 2) [23]. This pathway represents a series of alternating desaturation and elongation reactions that progressively introduce double bonds and extend the carbon chain of ALA (Figure 1) to yield EPA as the primary ω-3 product [12]. The pathway initiates with Δ6-desaturation of ALA to stearidonic acid (SDA, 18:4Δ6,9,12,15) catalyzed by Δ6-DES (EC 1.14.19.3; GenBank: AQX92135.1; Table 1, Figure 2) [9]. This front-end desaturase introduces a double bond at the Δ6 position (counting from the carboxyl terminus), converting the 18:3 substrate to an 18:4 intermediate [24]. Δ6-DES is an endoplasmic reticulum (ER) membrane-bound enzyme requiring molecular oxygen, cytochrome b5, and NADH as cofactors (Figure 2) (see Supplementary Information). In *N. oceanica* CCMP531/1779, the characterized Δ6-DES gene encodes a protein exhibiting high expression levels under standard cultivation conditions (Table 1), consistent with the organism’s robust capacity for ω-3 biosynthesis [9]. The 18:4 product undergoes C18→C20 elongation catalyzed by Δ6-elongase (ELOVL, EC 2.3.1.199; GenBank: AQX92136.1; Table 1, Figure 2), which adds a two-carbon unit to the carboxyl end of the acyl chain, yielding eicosatetraenoic acid (ETA, 20:4Δ8,11,14,17) [9]. Elongation proceeds through a four-step cycle involving condensation with malonyl-CoA (the rate-limiting step), reduction, dehydration, and a second reduction, collectively adding C_2_H_4_ while shifting double-bond positions by two carbons relative to the carboxyl terminus [25]. The Δ6-elongase, like the desaturase, is ER-localized and shows high constitutive expression in *N. oceanica* (Table 1) [9]. The critical final step in EPA biosynthesis is Δ5-desaturation of ETA to EPA (20:5Δ5,8,11,14,17), catalyzed by Δ5-desaturase (Δ5-DES, EC 1.14.19.37; GenBank: AQX92137.1; Table 1, Figure 2) [9]. This desaturase introduces a double bond at the Δ5 position, generating the characteristic five-double-bond structure of EPA (Figure 1 and Figure 2) [1,12]. Δ5-DES exhibits high catalytic activity in *N. oceanica*, ensuring efficient conversion of the C20:4 precursor to the C20:5 product [9]. The robust expression and activity of Δ5-DES are central to the EPA-rich phenotype of this organism (Figure 2, Table 1) [9,14]. The characterized biosynthetic genes (AQX92135.1, AQX92136.1, and AQX92137.1) were identified through genomic and transcriptomic analyses of *N. oceanica* strain CCMP531/1779 (Table 1) [9]. These genes represent the core ω-3 EPA biosynthesis machinery, with expression levels and enzymatic activities directly determining cellular EPA content [9]. Notably, upstream desaturases—Δ9-desaturase (GenBank: AQX92133.1) and Δ12-desaturase (GenBank: AQX92134.1)—generate oleic acid (C18:1) and linoleic acid (C18:2) precursors that, following ω-3 desaturation, feed into the ALA pool (Table 1) [9].

### 4.2. Regulation of EPA Biosynthesis, Metabolic Fate, and Turnover in N. oceanica

EPA biosynthesis in *N. oceanica* is subject to environmental and metabolic regulation, enabling adaptive modulation of membrane lipid composition and energy storage (Figure 2). Temperature exerts a particularly strong influence: cultivation at lower temperatures (15–20 °C versus 25–30 °C) upregulates desaturase expression and activity, increasing EPA content in both polar lipids and neutral lipids. This adaptive response maintains membrane fluidity under cold stress, a classic homeoviscous adaptation wherein increased PUFA incorporation counteracts temperature-induced rigidification [26]. Nitrogen availability profoundly affects lipid metabolism and EPA distribution. Nitrogen-replete conditions favor cell growth and membrane synthesis, with EPA predominantly incorporated into phospholipids to support expanding membrane systems. Nitrogen deprivation triggers a metabolic shift toward triacylglyceride (TGA) accumulation in cytoplasmic lipid droplets, increasing total lipid content (up to 40–60% dry cell weight) [27]. Under nitrogen starvation, EPA percentage in the total FA pool may decrease modestly as saturated and mono-unsaturated FAs accumulate in storage lipids, though absolute EPA content per cell often increases [27]. This subcellular partitioning of EPA—enrichment in phospholipids versus TAG—depends on nitrogen status and cultivation phase, with implications for extraction and bioavailability (see Supplementary Information). Light intensity and quality influence photosynthetic carbon fixation rates and, consequently, lipid biosynthesis flux. High light intensities can stimulate total lipid accumulation but may also induce photoinhibition and oxidative stress, potentially affecting desaturase activities. The interplay between light-driven carbon assimilation, nitrogen-dependent growth regulation, and temperature-modulated desaturase expression creates a complex regulatory landscape that can be manipulated for optimized EPA production in industrial cultivation systems.

At the molecular level, transcriptional regulation of desaturase and elongase genes likely involves transcription factors responsive to temperature, lipid intermediates, and cellular energy status, though these regulatory networks remain incompletely characterized in *N. oceanica*. Post-transcriptional mechanisms—including mRNA stability, translational control, and enzyme turnover—may also contribute to dynamic EPA biosynthesis regulation. Elucidating these control mechanisms represents a key frontier for rational metabolic engineering to enhance EPA yields. Once synthesized and incorporated into cellular lipids, EPA undergoes several metabolic fates. In membrane phospholipids, EPA is relatively stable with slow turnover (half-life of days to weeks in mammalian systems; comparable data for *N. oceanica* are limited). Phospholipid-esterified EPA serves dual roles: maintaining membrane biophysical properties and serving as a substrate reservoir for eicosanoid biosynthesis following phospholipase A_2_-mediated release (Figure 3) [28]. EPA-containing TAGs represent energy storage that can be mobilized during periods of nutrient limitation or darkness when photosynthesis ceases. Lipase-mediated hydrolysis releases free fatty acids, including EPA, which undergo β-oxidation in peroxisomes and mitochondria to generate acetyl-CoA for energy production or biosynthetic precursors [29]. Peroxisomal β-oxidation preferentially degrades very-long-chain and PUFAs, while mitochondrial β-oxidation processes shorter and less unsaturated species, creating compartmentalized degradation pathways [30].

In summary, the well characterized genetic architecture of the EPA biosynthesis pathway in *N. oceanica* (Table 1, Figure 2)—with well-defined Δ6-DES, Δ6-elongase, and Δ5-DES genes—provides a foundation for future metabolic engineering efforts to enhance EPA yields, redirect carbon flux, or engineer novel FA profiles. Understanding the biosynthetic origins and metabolic fate of EPA in this model organism bridges the molecular foundation with the mechanistic understanding of the bioactivity and clinical applications of EPA.

## 5. Molecular Mechanisms of Action

The molecular versatility of EPA—existing in multiple esterified forms, undergoing controlled enzymatic transformations, and serving as both structural component and signaling precursor—positions this fatty acid as a central node in lipid metabolism and cellular signaling networks. Understanding EPA’s chemical structure (Figure 1) and physicochemical properties provides the foundation for elucidating enzymatic transformations (Figure 3, Table 2), whole-organism pharmacology, and clinical applications.

### 5.1. Membrane Integration and Biophysical Effects

Quantitatively, EPA comprises 1–3% of total FAs in typical Western diet consumers but can reach 5–10% in individuals consuming high levels of marine ω-3s or *N. oceanica*-derived supplements [31]. The spatial arrangement of double bonds in EPA directly determines its biological functionality through multiple mechanisms. In biological systems, EPA rarely exists as the free (unesterified) fatty acid. Instead, it is predominantly esterified into complex lipids that define its subcellular localization and metabolic fate [32]. The primary fate of dietary or endogenously synthesized EPA is incorporation into the sn-2 position of membrane phospholipids (PL) [33]. The incorporation of EPA into membrane PLs is mediated by acyl-CoA synthetases (activating EPA to EPA-CoA), lysophospholipid acyltransferases (esterifying EPA-CoA to lysophospholipids), and phospholipid remodeling enzymes of the Lands cycle [32]. Once integrated, EPA physically displaces other fatty acids—particularly ARA (20:4n-6)—from membrane phospholipids, a competitive process governed by relative abundance, enzyme substrate preferences, and acyl-CoA pool dynamics [28].

EPA-enriched membranes exhibit increased fluidity compared to membranes enriched in saturated or mono-unsaturated fatty acids and even compared to ARA-enriched membranes despite both being polyunsaturated. As stated previously, the additional double bond at Δ17 in EPA (Figure 1 and Figure 3) introduces further conformational disorder, loosening acyl chain packing and expanding the lateral area per lipid molecule. This increased fluidity influences membrane protein function through multiple mechanisms: altered protein conformational equilibria, modified lateral diffusion rates affecting protein–protein interactions, and changes in lipid raft organization, the cholesterol- and sphingomyelin-enriched microdomains that compartmentalize signaling molecules [34]. These membrane perturbations influence the conformation, oligomerization, and activity of integral membrane proteins including ion channels, G-protein-coupled receptors, and receptor tyrosine kinases [35]. Membrane PL EPA also serves as a reservoir for eicosanoid biosynthesis upon phospholipase A_2_-mediated release [28].

Lipid raft modulation by EPA has functional significance (see Supplementary Information). EPA incorporation disrupts raft stability and alters the partitioning of raft-associated proteins including Src-family kinases, G-protein coupled receptors, and Toll-like receptor 4 (TLR4) [36]. Displacement of signaling proteins from lipid rafts can attenuate inflammatory signaling cascades; for example, EPA-mediated disruption of TLR4 raft localization reduces lipopolysaccharide (LPS)-induced inflammatory responses in macrophages [36]. Conversely, EPA can promote the formation of alternative lipid domains enriched in specific signaling complexes, creating spatially organized signaling platforms distinct from classical cholesterol-sphingomyelin rafts.

Beyond bulk membrane properties, EPA influences specific membrane protein functions through direct lipid–protein interactions [35]. Ion channels, including voltage-gated sodium channels, L-type calcium channels, and various potassium channels, exhibit altered gating kinetics, conductance, or pharmacological sensitivity in EPA-enriched membranes [37]. These effects likely arise from EPA molecules directly interacting with hydrophobic protein domains, altering the energetics of conformational transitions [38]. Such mechanisms contribute to the reported anti-arrhythmic properties of EPA, wherein modulation of cardiac ion channel function stabilizes membrane electrical activity [38].

### 5.2. Competitive Enzymatic Metabolism: The ARA-EPA Axis

A central mechanism underlying EPA’s biological activity is competitive substrate metabolism with ARA (Figure 3, Table 2) [11]. Both EPA and ARA are substrates for the same families of oxygenase enzymes—prostaglandin synthesases (PTGS, formerly designated as COX), lipoxygenases (ALOX, formerly designated as LOX), and cytochrome P450 enzymes—but yield metabolite products with profoundly different, often opposing biological activities [39]. Enzymatic oxidation products represent functionally critical EPA derivatives (Figure 3, Table 2), PTGS-derived 3-series prostaglandins and thromboxanes [40], lipoxygenase-derived leukotrienes and resolvins [7], and cytochrome P450-derived epoxides and hydroxides [41] that constitute a structurally diverse library of bioactive mediators. Non-enzymatic oxidation generates isoprostanes—prostaglandin-like compounds formed via free radical-catalyzed mechanisms—which serve as biomarkers of oxidative stress but may also exert biological activities. The specific positioning of double bonds governs substrate recognition by biosynthetic enzymes [39].

*Prostaglandin synthesase pathway:* PTGS1 and PTGS-2 catalyze the committed step in prostaglandin and thromboxane biosynthesis, converting 20-carbon PUFAs to prostanoid endoperoxide intermediates (PGG_2_ from ARA; PGG_3_ from EPA), which are subsequently reduced to PGH_2_ or PGH_3_ and then converted to specific prostaglandins and thromboxanes by downstream synthases (Figure 3) [40,42]. PTGS-1 and PTGS-2 catalyze bis-oxygenation of EPA to generate prostaglandin H_3_ (PGH_3_), the precursor to 3-series prostaglandins and thromboxane A_3_ (Figure 3) [40]. Critically, the Δ17 double bond unique to EPA (Figure 3) sterically hinders productive enzyme–substrate alignment, resulting in slower turnover rates and reduced product formation compared to ARA metabolism [43]. ARA-derived prostanoids include prostaglandin E_2_ (PGE_2_), a potent mediator of inflammation, pain, and fever but also a pleiotropic mediator dependent on type of E2 receptors present on target cells [44]; prostacyclin I_2_ (PGI_2_), a vasodilator and platelet inhibitor but pro-inflammatory in certain contexts; and thromboxane A_2_ (TXA_2_), a powerful platelet aggregator and vasoconstrictor (Figure 3) [45]. The pleiotropic role of PGE_2_ should be taken into account in clinical contexts where tissue repair is the primary concern, as it drives tissue regeneration in the skin, bone, heart, intestine, liver, and kidney through stem cell proliferation, M2 macrophage polarization, angiogenesis, and extracellular matrix remodeling through E-type prostenoid receptor signaling [46].

EPA-derived prostanoids: Production relies on the liberation of EPA from the sn-2 position of membrane phospholipids (PLs) by phospholipase A_2_ (PLA_2_). Specific PLA_2_ isoforms (calcium-independent PLA_2_ (iPLA_2_) and secreted PLA_2_ (sPLA)) prefer EPA over ARA, while cytosolic PLA_2_ (cPLA_2_) is more effective for ARA, yielding only 10% liberation of EPA from the sn-2 position of PLs [47]. The PTGS generated 3-series prostaglandins (PGE_3_ and PGI_3_) and thromboxane A_3_ (TXA_3_) are structurally analogous but functionally attenuated (Figure 3; Table 2) [40,48]. PGE_3_ exhibits substantially reduced inflammatory potency compared to PGE_2_, binding prostaglandin E receptors (EP1–EP4) with lower affinity and producing weaker downstream signaling [43]. Critically, TXA_3_ is virtually inactive as a platelet aggregator and vasoconstrictor relative to TXA_2_ (Table 2), with >50-fold reduced potency in functional assays [49]. This difference arises from subtle conformational variations affecting receptor binding and explains the anti-thrombotic effects of EPA: displacement of ARA from membrane stores and preferential PTGS metabolism of EPA shifts prostanoid production from the pro-thrombotic, pro-inflammatory 2-series to the minimally active 3-series. Importantly, EPA competes with ARA for PTGS enzyme active sites [43]. While PTGS enzymes can metabolize both substrates, kinetic studies reveal K_m_ values and V_max_ differences that favor ARA under equimolar conditions [43]. However, when EPA is present in significant molar excess—achievable through dietary supplementation —it effectively competes for PTGS binding, reducing ARA metabolism even if EPA itself is metabolized more slowly [50]. The net effect is a reduction in bioactive 2-series prostanoid production, with EPA serving as both a competitive inhibitor of ARA metabolism and a precursor to less potent 3-series products [11].

*Lipoxygenase pathway:* 5-lipoxygenase (ALOX-5), expressed primarily in leukocytes, catalyzes the first committed step in leukotriene biosynthesis [51]. ARA is converted via 5-hydroperoxyeicosatetraenoic acid (5-H_P_ETE) to leukotriene A_4_ (LTA_4_), which is then metabolized to leukotriene B_4_ (LTB_4_)—a potent neutrophil chemo-attractant—or conjugated with glutathione to form cysteinyl leukotrienes (LTC_4_, LTD_4_, and LTE_4_) involved in broncho-constriction and allergic responses (Figure 3) [52]. Similarly, ALOX-5 converts EPA to leukotriene A_5_ (LTA_5_) and subsequently leukotriene B_5_ (LTB_5_), which exhibits 10–100-fold lower biological potency than the ARA-derived leukotriene B_4_ (LTB_4_; Table 2) due to subtle conformational differences affecting receptor binding affinity [53]. The molecular basis for this attenuation lies in the additional double bond at Δ17 in the EPA-derived product (Figure 1), which subtly alters the three-dimensional structure and reduces affinity for the BLT1 receptor (the high-affinity LTB_4_ receptor) (see Supplementary Information). Consequently, EPA supplementation reduces inflammatory cell recruitment by displacing ARA from ALOX-5 metabolism and generating the weakly active LTB_5_ rather than the potent LTB_4_.

ALOX-12 and ALOX-15 pathways also metabolize both ARA and EPA, generating hydroxyeicosatetraenoic acids (HETEs from ARA) and hydroxyeicosapentaenoic acids (HEPEs from EPA) with diverse biological activities [54]. Of particular significance in humans, ALOX-15b metabolism of EPA yields primarily 15-hydroxyeicosapentaenoic acid (15-HEPE), a key intermediate in specialized pro-resolving mediator biosynthesis [55,56]. The Δ17 double bond is essential for ALOX-15-mediated or aspirin-acetylated PTGS-2-mediated hydroxylation at carbon-15, yielding 15-HEPE, the committed intermediate for resolvin E biosynthesis [55]. Neither ARA nor DHA can substitute for EPA in this pathway, underscoring the unique structure-function relationship whereby a single double-bond position dictates access to an entire class of bioactive lipids [7]. Selective oxidation of bis-allylic positions generates regio- and stereoisomeric hydroperoxide and hydroxide derivatives with distinct biological activities [54]. For example, 5-hydroxyeicosapentaenoic acid (5-HEPE) and 12-HEPE exhibit chemotactic and inflammatory modulating properties, while 15-HEPE serves as a resolvin precursor [56]. The multiplicity of potential oxidation sites—dictated by double-bond positioning—endows EPA with the capacity to generate a diverse array of oxylipins, each with specific receptor targets and functional outcomes [57].

*Cytochrome P450 pathway:* Epoxygenases (CYP2C and CYP2J Cyt P450 subfamilies) convert ARA to epoxyeicosatrienoic acids (EETs) and EPA to epoxyeicosapentaenoic acids (EpEPEs or EEQs), while ω-hydroxylases generate 20-HETE from ARA and analogous products from EPA (Figure 3) [41]. While Cyp1A1, Cyp2C8, Cyp2C9, and Cyp2J2 epoxidize ARA, EPA, and DHA, Cyp2J2 appears to be the main epoxidase for EPA [58]. EETs generally exhibit vasodilatory, anti-inflammatory, and cardioprotective properties, though effects are context-dependent [41]. EpEPEs derived from EPA show similar or enhanced protective activities, contributing to cardiovascular benefits. The precise structure-activity relationships of these metabolites remain an active area of investigation.

### 5.3. Specialized Pro-Resolving Mediators: Resolvins

A mechanistically distinct and clinically significant pathway unique to EPA is the biosynthesis of E-series resolvins (RvE1, RvE2, and RvE3), which are specialized pro-resolving mediators (SPMs) that actively promote the resolution of inflammation (Figure 3; Table 2) [7,59]. Unlike passive inflammation decay or immunosuppression, resolution is an active, coordinated process involving cessation of neutrophil infiltration, apoptosis of inflammatory cells, efferocytosis (macrophage clearance of apoptotic cells), tissue repair, and return to homeostasis [60]. Resolvins are enzymatically generated lipid mediators that orchestrate these resolution programs [3,61], but effective clinical therapies face several major challenges, such as very short systemic half-lives and therefore therapeutic durations, patient-dependent conversion of dietary fish oil-derived EPA due to factors like genetics, aging, poor diet-induced metabolic bottlenecks, and potential drug interferences, e.g., non-steroidal anti-inflammatory drugs can actively block the PTGS pathway necessary for resolvin production [62,63,64]. Resolvin E biosynthesis begins with EPA oxygenation by ALOX-15 (in humans) or aspirin-acetylated PTGS-2 to yield 15R-hydroxyeicosapentaenoic acid (15R-HEPE) or 15S-HEPE, respectively [55]. These intermediates undergo further ALOX-5-catalyzed transformation to generate RvE1 (5S,12R,15R-trihydroxy-EPA) and RvE2 (5S,15R-dihydroxy-EPA), with RvE3 arising through alternative oxygenation patterns [65]. The stereochemistry of hydroxyl group positions is critical for receptor recognition and biological activity [55]. RvE1, the most extensively characterized E-series resolvin, exerts potent anti-inflammatory and pro-resolving activities at nanomolar concentrations through receptor-mediated mechanisms [66]. RvE1 binds the ChemR23 receptor (also known as ERV1 or CMKLR1), a G-protein-coupled receptor expressed on macrophages, dendritic cells, and adipocytes, initiating signaling cascades that enhance efferocytosis, reduce inflammatory cytokine production, and promote anti-inflammatory cytokine release. RvE1 also acts as a partial agonist/antagonist at the BLT1 receptor (the LTB_4_ receptor), blocking LTB_4_-mediated neutrophil chemotaxis and adhesion without triggering full agonist responses [55]. This dual receptor engagement—activating pro-resolving pathways via ChemR23 while blocking pro-inflammatory pathways via BLT1—exemplifies the sophisticated pharmacology of resolvins [67].

Critically, resolvins have no ARA-derived counterparts (Table 2) [7], as the Δ17 double bond in EPA (Figure 3) is essential for the enzymatic transformations leading to resolvins [55]. This represents a qualitatively unique mechanism of action for EPA: not merely producing less potent versions of ARA-derived mediators (as with prostaglandins and leukotrienes) but generating entirely novel signaling molecules with distinct, resolution-promoting biological activities. This mechanistic uniqueness underpins the utility of EPA in inflammatory disease management and distinguishes it from ARA, saturated fatty acids, and even DHA (which generates distinct D-series resolvins, protectins, and maresins through separate pathways) [54].

### 5.4. Receptor-Mediated Signaling Independent of Enzymatic Metabolism

Beyond serving as substrates for enzymatic transformation, EPA and its metabolites engage specific receptors that directly modulate cellular signaling pathways [68]. The free FA receptor 4 (FFA4, also known as GPR120), a G-protein-coupled receptor expressed in adipocytes, macrophages, and intestinal cells, is activated by long-chain ω-3 EFAs including EPA [69]. FFA4 activation by EPA triggers anti-inflammatory signaling through β-arrestin-2-mediated pathways, inhibiting TAK1 (transforming growth factor-β-activated kinase 1) and downstream NF-κB and JNK inflammatory cascades [68]. This receptor-mediated mechanism contributes to the anti-inflammatory effects of EPA in obesity and metabolic syndrome, independent of eicosanoid biosynthesis [70].

Peroxisome proliferator-activated receptors (PPARs), ligand-activated transcription factors of the nuclear receptor superfamily, are also targets of EPA [71]. PPARα, predominantly expressed in the liver, heart, and skeletal muscle, is activated by EPA and EPA-CoA, inducing the transcription of genes involved in FA β-oxidation, ketogenesis, and lipoprotein metabolism [72]. This contributes to TAG-lowering effects of EPA. PPARγ, expressed in adipose tissue and immune cells, is modestly activated by EPA, promoting adipocyte differentiation and insulin sensitization while suppressing inflammatory gene expression in macrophages. The interaction of EPA with PPARs is direct (EPA or EPA-CoA binds the ligand-binding domain) and results in transcriptional reprogramming favoring lipid catabolism and anti-inflammatory gene expression (see Supplementary Information).

EPA and its oxidized metabolites also modulate NF-κB signaling—a master regulator of inflammatory gene expression—through multiple mechanisms beyond FFA4 [50]. EPA inhibits IκB kinase (IKK) activity, reducing phosphorylation and degradation of IκB inhibitory proteins and thereby preventing NF-κB nuclear translocation [50]. EPA-derived resolvins and other SPMs further suppress NF-κB through receptor-mediated signaling [61]. Additionally, EPA modulates sterol regulatory element-binding protein (SREBP) pathways, particularly SREBP-1c, a transcription factor controlling lipogenic enzyme expression [73]. EPA reduces SREBP-1c nuclear abundance and transcriptional activity, thereby decreasing hepatic lipogenesis and contributing to the TAG lowering effect.

### 5.5. Mitochondrial and Cellular Energetic Effects

EPA is also incorporated into TAGs within lipid droplets, particularly following high-dose supplementation or in adipose tissue. In *N. oceanica*, EPA-enriched TAGs serve as energy storage molecules, with EPA comprising 30–50% of total FAs under optimal cultivation conditions (Figure 2, Table 1) [9]. Cholesteryl esters containing EPA are minor components of lipoprotein particles, contributing to their physicochemical properties and susceptibility to oxidation. EPA influences cellular energy metabolism and mitochondrial function through multiple pathways. As a substrate for β-oxidation (see below), EPA generates acetyl-CoA that feeds into the tricarboxylic acid (TCA) cycle, supporting ATP production [29]. Peroxisomal β-oxidation preferentially processes very-long-chain and highly unsaturated fatty acids like EPA, chain-shortening them to medium-chain products that undergo complete mitochondrial oxidation [30,74].

Beyond serving as fuel, EPA modulates mitochondrial biogenesis and function [75]. EPA activates AMP-activated protein kinase (AMPK), a cellular energy sensor, through mechanisms involving altered AMP:ATP ratios or direct/indirect effects on upstream kinases [76]. AMPK activation phosphorylates and activates PGC-1α (peroxisome proliferator-activated receptor γ coactivator-1α), a master regulator of mitochondrial biogenesis, increasing mitochondrial mass, oxidative capacity, and metabolic efficiency [77]. This mechanism contributes to effects of EPA on metabolic health and insulin sensitivity [75]. EPA incorporation into mitochondrial membrane PLs—particularly cardiolipin, the signature phospholipid of the inner mitochondrial membrane—modulates respiratory chain function, membrane potential, and reactive oxygen species (ROS) generation [78]. EPA-enriched cardiolipin may enhance electron transport chain efficiency or alter ROS production kinetics, with potential implications for oxidative stress, aging, and degenerative diseases, though these effects remain mechanistically complex and context-dependent [78].

### 5.6. Integration of Mechanisms: The ARA:EPA Ratio

The molecular mechanisms described above operate concurrently and interactively, with the balance between EPA-derived and ARA-derived signaling determined primarily by the tissue ARA:EPA ratio [8,79]. High ARA:EPA ratios (>5:1, typical of Western diets with low ω-3 intake) result in predominant generation of pro-inflammatory, pro-thrombotic eicosanoids with minimal resolvin production (Figure 3) [80]. Conversely, low ARA:EPA ratios (<3:1, achievable through regular consumption of marine ω-3s or *N. oceanica*-biomass/oil or krill-derived EPA supplements) shift eicosanoid production toward less inflammatory 3-series prostaglandins and 5-series leukotrienes while enabling robust resolvin biosynthesis (Figure 3; Table 2) [81]. This biochemical shift translates to reduced inflammatory tone, enhanced resolution capacity, decreased thrombotic potential, and improved metabolic regulation [82]. Optimal ratios (1:1 to <1:1) indicate EPA-predominant membrane phospholipid composition, where EPA-derived eicosanoids numerically and functionally outcompete ARA-derived mediators without reducing or depleting ALA pools (noticeably most of dietary ALA is oxidized due to inadequate n-3 conversion in human metabolism or the levels of linoleic acid (LA) in mice skin [83,84]). The ARA:EPA ratio in plasma PLs or erythrocyte membranes serves as a biochemical index of this competitive balance, integrating dietary intake, endogenous biosynthesis, and tissue incorporation kinetics into a single quantitative biomarker. Mechanistically, lowering the ARA:EPA ratio through EPA supplementation represents a multifaceted intervention: competitive displacement of ARA from membranes, reduced generation of potent ARA-derived inflammatory mediators, increased production of weakly active EPA-derived eicosanoids, and enabled biosynthesis of unique pro-resolving resolvins [11]. These synergistic mechanisms explain why EPA supplementation effects exceed simple dilution of ARA and why the ARA:EPA ratio exhibits dose–response relationships with clinical outcomes [71], although patient-specific applications should be reviewed in light of potential hemolytic activities of EPA through increased membrane fluidity, an effect (as explained earlier) not necessarily due to incorporation of EPA into erythrocyte membranes.

## 6. Pharmacological Profile: Pharmacology, Pharmacokinetics, and Clinical Applications

The translation of EPA’s molecular mechanisms into physiological and clinical effects depends critically on its absorption, distribution, metabolism, and elimination, collectively defining its pharmacokinetic profile. Understanding EPA pharmacology synthesizes the molecular foundation with clinical applications and informs optimal dosing strategies for achieving therapeutic tissue levels.

### 6.1. Absorption, Distribution, Tissue Incorporation, Bioavailability, Metabolism and Biotransformation, Elimination, and Clearing of EPA

**Figure 4:**
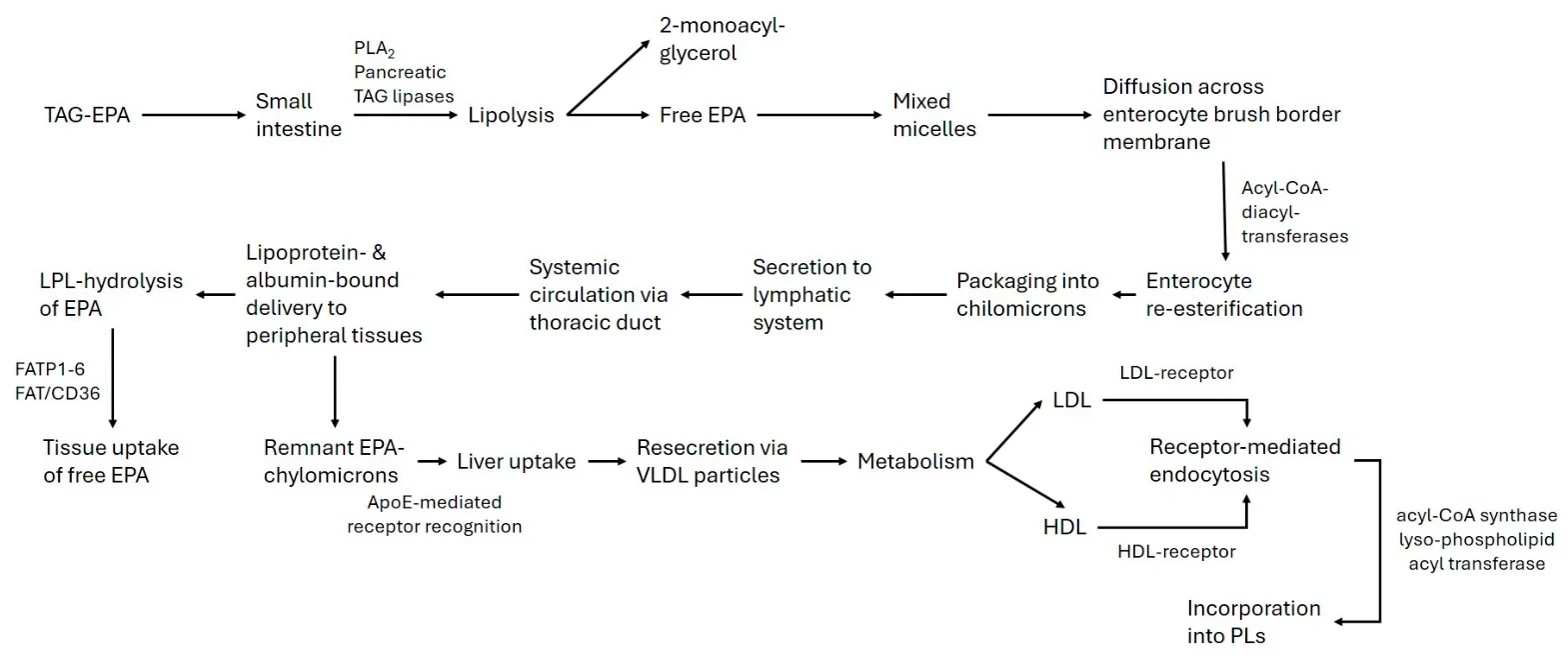
Uptake and incorporation of EPA into phospholipids. apoE, apolipoprotein E; EPA, eicosapentaenoic acid; FATP1-6, fatty acid transport proteins 1-6; FAT/CD36, fatty acid translocase/cluster of differentiation 36; HDL, high-density lipoprotein; LDL, low-density lipoprotein; LPL, lipoprotein lipase; PLA_2_, phospholipase A_2_; TAG triacylglyceride; VLDL, very-low-density lipoprotein.

Dietary EPA, whether from marine sources or *N. oceanica*-derived supplements, is consumed primarily in esterified forms—predominantly as TAGs in fish—and algal oil, or as PLs in krill oil [85]. The chemical form profoundly influences absorption efficiency and bioavailability [86]. In mammalian systems (relevant for *N. oceanica*-derived EPA), dietary EPA is absorbed in the small intestine (Figure 4). In the gastrointestinal tract, esterified EPA undergoes lipolysis by pancreatic lipases (primarily pancreatic TAG lipase and phospholipase A_2_), releasing free EPA and 2-mono-acylglycerols [87]. Free EPA is incorporated into mixed micelles formed with bile salts, PLs, and cholesterol, facilitating diffusion across the enterocyte brush border membrane (Figure 4). Within enterocytes, EPA is rapidly re-esterified into TAGs by acyl-CoA:diacylglycerol acyltransferases and packaged into chylomicrons for secretion into the lymphatic system, bypassing first-pass hepatic metabolism. Chylomicrons enter the systemic circulation via the thoracic duct, delivering EPA to peripheral tissues (Figure 4 and see Supplementary Information). Following chylomicron-mediated delivery, EPA is distributed to tissues via multiple lipoprotein-mediated and albumin-bound pathways. Lipoprotein lipase (LPL) on endothelial surfaces hydrolyzes chylomicron TAGs, releasing EPA for tissue uptake (Figure 4). Remnant chylomicrons, enriched in EPA-containing lipids, are taken up by the liver via apoE-mediated receptor recognition. Hepatic EPA is re-secreted in VLDL particles, further metabolized to LDL and HDL, creating a dynamic plasma lipoprotein EPA pool with a circulating half-life of 48–72 h [28]. Tissue uptake of lipoprotein-associated EPA occurs via receptor-mediated endocytosis (LDL receptor and VLDL receptor) and through FA transport proteins (FATP1-6) and FA translocase (FAT/CD36) (Figure 4) that facilitate transmembrane flux of free FAs released by lipoprotein lipase [88]. Tissue-specific expression of these transporters, combined with local metabolic demand, determines EPA distribution patterns [88]. EPA incorporation into tissue PLs follows a biphasic pattern [17]. Initial rapid incorporation (within 24–48 h) reflects EPA uptake and esterification into PLs by membrane remodeling enzymes (acyl-CoA synthetase, lysophospholipid acyltransferases) [33] (Figure 4). Steady-state incorporation, achieved after several weeks of consistent intake, depends on dose, baseline ARA levels, and tissue-specific turnover rates [89]. Cardiac tissue, liver, and blood cells (erythrocytes, platelets, and leukocytes) exhibit avid EPA incorporation, with steady-state EPA content reaching 3–8% of total FAs at typical supplemental doses (1–2 g/day) [31]. Brain tissue shows limited EPA accumulation (<1% of brain fatty acids) despite chronic supplementation, reflecting blood–brain barrier selectivity that preferentially transports DHA over EPA [90]. This contrasts sharply with DHA, which accumulates to 10–15% of brain PL FAs, explaining differential neurological effects of EPA versus DHA [90]. Adipose tissue serves as a long-term EPA reservoir, with adipocyte TAG stores reflecting chronic dietary intake patterns [4]. Adipose EPA content correlates with dietary consumption over months to years, providing a biomarker of long-term ω-3 status [91]. However, adipose EPA mobilization during lipolysis is slow relative to circulating turnover, limiting adipose stores as an acute EPA source during dietary deprivation [91].

Bioavailability—the fraction of ingested EPA reaching systemic circulation—varies with formulation and food matrix. Re-esterified TAGs (rTAG), the predominant form in most fish oils and *N. oceanica* products, exhibit bioavailability of approximately 60–70% under fasted conditions, increasing to 80–90% when consumed with a fat-containing meal due to enhanced lipase secretion and micelle formation [86]. Ethyl ester (EE) formulations, common in pharmaceutical ω-3 preparations, show reduced bioavailability (40–50% fasted) because ethyl ester hydrolysis by carboxyl ester lipase is less efficient than TAG hydrolysis; co-administration with dietary fat improves EE bioavailability to ~70% [85]. Phospholipid-bound EPA (PL-EPA), as in krill oil, demonstrates comparable or slightly superior bioavailability to rTAG-EPA, potentially due to enhanced emulsification properties and lysophospholipid-mediated absorption pathways [13]. Free fatty acid (FFA) formulations exhibit the highest bioavailability (>90%) as they bypass lipolysis, though FFA-EPA is rarely used due to stability and palatability concerns.

*Nannochloropsis oceanica*-derived EPA, delivered as TAG-esterified oil, exhibits bioavailability characteristics comparable to fish oil rTAG formulations. The high EPA content (30–50% of total FAs) and minimal DHA content (<2%) result in predominant EPA delivery without competition from DHA for incorporation pathways [9,14]. Comparative bioavailability studies between algal-sourced EPA and fish-sourced EPA show equivalent or superior absorption kinetics for algal products, validating *N. oceanica* as a functionally equivalent alternative to marine animal sources.

The quantitatively dominant metabolic fate is β-oxidation in mitochondria and peroxisomes, yielding acetyl-CoA for energy production or biosynthetic precursors [29,30]. Peroxisomal β-oxidation preferentially processes EPA and other long-chain polyunsaturated fatty acids, chain-shortening EPA by sequential removal of two-carbon units [74]. Shortened products undergo complete mitochondrial oxidation [29]. Under conditions of high EPA intake, β-oxidation rates increase, maintaining EPA homeostasis and preventing excessive accumulation. As discussed previously, EPA undergoes extensive metabolism via multiple pathways, including β-oxidation and enzymatic oxygenation pathways (PTGS, ALOX, and cytochrome P450-mediated transformations), converting EPA from a relatively inert storage molecule into potent signaling mediators that mediate anti-inflammatory, pro-resolving, and cardioprotective effects [3,7,61]. The kinetics of these transformations are influenced by enzyme expression levels, cofactor availability (NADH and O_2_), and competitive inhibition by ARA [39].

EPA elimination occurs primarily via β-oxidation to CO_2_ and H_2_O, with exhaled CO_2_ representing the ultimate excretion route for the carbon skeleton. Urinary excretion of intact EPA is negligible due to high lipophilicity (log P ~6.8) and extensive protein binding in plasma [16]. Biliary excretion of EPA-containing PLs and oxidized metabolites contributes minimally to overall elimination but plays a role in enterohepatic recirculation. The apparent terminal half-life of EPA in plasma PLs is approximately 48–72 h following acute dosing, though steady-state kinetics during chronic supplementation are better described by tissue-specific turnover rates. Erythrocyte EPA, a commonly measured biomarker, exhibits a half-life corresponding to red blood cell lifespan (~120 days), providing an integrated measure of EPA status over several months [92]. Platelet EPA turns over more rapidly (~10 days), reflecting platelet lifespan and making platelet PL-EPA a sensitive marker of recent intake changes. Clearance rates are dose-dependent and saturable. At low doses (<500 mg/day), EPA incorporation into tissues is highly efficient with minimal β-oxidative loss. At high doses (>3 g/day), β-oxidation increases proportionally, limiting tissue accumulation and creating a plateau effect wherein further dose escalation yields diminishing returns in tissue EPA content [4]. These saturation kinetics inform dosing strategies: moderate doses (1–2 g/day) achieve favorable tissue incorporation, while very high doses (>4 g/day) used in specific clinical applications (e.g., severe hypertriglyceridemia) rely on overwhelming β-oxidative capacity to achieve supraphysiological tissue levels [93]. As with all dietary supplementary interventions, you can have too much of a good thing in certain clinical circumstances. The above does not account for peroxidation of EPA in patients with compromised antioxidant capacity. Here, high doses of EPA may induce non-enzymatic peroxidation of EPA, generating F3-isoprostanes and other oxylipins and neuroprostanes. A Fenton reaction forms a hydroxyl EPA radical that reacts with molecular oxygen to form a peroxyl radical. This peroxyl radical can attack neighboring membrane PUFAs, thereby propagating hydroxyl radical formation in the membrane, with adverse effects on membrane fluidity and potential induction of cell death pathways [94]. This potential side effect of high EPA supplementation highlights that formulations need to be personalized to meet a patient’s need and to counteract any adverse effects of supplementation.

### 6.2. Dose–Response Relationships, Therapeutic Targeting, Drug Interactions, and Safety Pharmacology

The relationship between EPA dose and biological/clinical effects exhibits complex, non-linear dynamics reflecting pharmacokinetic saturation, competitive displacement of ARA, and threshold effects for biomarker changes) (see Supplementary Information). Plasma EPA concentration increases dose-dependently up to ~3–4 g/day, beyond which incremental increases diminish due to enhanced β-oxidation [95]. At 1 g/day supplementation, plasma pPL-EPA typically increases from baseline ~0.5–1% to 2–3% of total fatty acids. At 2–4 g/day, EPA reaches 5–8%, approaching parity with or exceeding ARA levels (typically 8–12% in Western populations) (see Supplementary Information). This dose range effectively lowers the ARA:EPA ratio from typical values of 5–15:1 to target ranges of 1–3:1, shifting eicosanoid production toward EPA-derived mediators [8,79].

Tissue-specific dose–response relationships vary. Cardiac tissue EPA shows near-linear dose-dependence up to ~2 g/day, plateauing thereafter [31]. Hepatic EPA accumulates more avidly, correlating with TAG-lowering effects [17]. Immune cell (neutrophil and monocyte) EPA incorporation demonstrates threshold effects: minimal functional changes occur below ~1% -PL-EPA, but marked reductions in pro-inflammatory mediator production emerge above 2–3% EPA, corresponding to ~1–2 g/day intake (see Supplementary Information). Time to steady state varies by tissue: plasma PLs achieve 90% of steady-state EPA within 2–4 weeks, erythrocytes require 3–4 months, and adipose tissue stabilizes over 6–12 months [89]. As mentioned earlier, EPA incorporation into membrane lipids alters membrane fluidity. A study using dietary supplementation of fish oil (930 mg EPA and 630 mg DHA) over 42 days showed a reduced ratio of membrane n-6 to total membrane lipids, reduced platelet aggregation in response to ADP, elevated levels of both PUFAs in cheek cells, and a sustained influence on membrane composition and fluidity, which were not necessarily due to increased membrane EPA or DHA [96]. The high purity EPA-rich oil void of DHA) simplifies dose–response interpretation by eliminating DHA-mediated effects that confound fish oil studies, positioning *N. oceanica* EPA as an ideal intervention for isolating EPA-specific pharmacology and clinical efficacy. On the other hand, a study using human erythrocytes and exposing them to anti-cancer concentrations of EPA (10–100 µM) showed hemolytic activity and eryptosis through calcium buildup and AChE inhibition, suggesting optimizing the anti-cancer potential of EPA by including calcium channel blockers and chelators to minimize toxicity to off-target tissue [97]. This highlights that dietary supplementation dosages should be individually assessed, as outcomes are potentially strongly influenced patient and clinical circumstances. Clinical trial designs must account for these kinetics; trials assessing biomarker changes can detect effects within 4–8 weeks, while trials measuring clinical endpoints (cardiovascular events and disease progression) require months to years for EPA tissue equilibration and mechanistic effects to manifest [79,98] (see Supplementary Information). Bioavailability of *N. oceanica*-derived EPA formulations has been evaluated in comparative studies showing dose-proportional increases in plasma and erythrocyte EPA, with kinetics indistinguishable from high-quality fish oil products.

EPA exhibits relatively few clinically significant drug interactions due to minimal effects on the cytochrome P450 enzyme systems. However, pharmacodynamic interactions warrant consideration. Anticoagulant and antiplatelet medications (warfarin, aspirin, and clopidogrel) may exhibit enhanced effects when combined with high-dose EPA (>2 g/day) due to additive anti-thrombotic mechanisms (see Supplementary Information). While this interaction is generally modest and clinically manageable, monitoring of bleeding parameters is advised in patients on combined therapy (see Supplementary Information), and patients and consumers should seek advice from healthcare professionals. Lipid-lowering agents, particularly statins and fibrates, show complementary rather than antagonistic interactions with EPA [95]. Combination therapy often produces additive TAG reduction and may enhance anti-inflammatory effects, though clinical trial evidence for superiority over monotherapy is mixed [93]. Antihypertensive medications may exhibit modest additive blood pressure reduction when combined with EPA, reflecting EPA’s vasodilatory effects mediated by prostacyclin I_3_ and nitric oxide modulation [99]. Safety pharmacology studies demonstrate excellent tolerability of EPA across a wide dose range [100]. Gastrointestinal effects (mild nausea and fishy aftertaste) are dose- and formulation-dependent, with enteric-coated and microencapsulated products reducing these symptoms. Clinically significant adverse effects are rare even at doses up to 4–6 g/day used in prescription formulations [101]. Prolongation of bleeding time occurs at high doses but rarely translates to clinically significant bleeding in the absence of co-administered anticoagulants [95]. Unlike earlier concerns, comprehensive clinical trials (REDUCE-IT and JELIS) demonstrate no increased bleeding risk requiring discontinuation at therapeutic EPA doses [5,6].

## 7. Genomic and Signaling Integration: Gene Expression and Signaling Pathways in Cellular and Clinical Context

**Figure 5:**
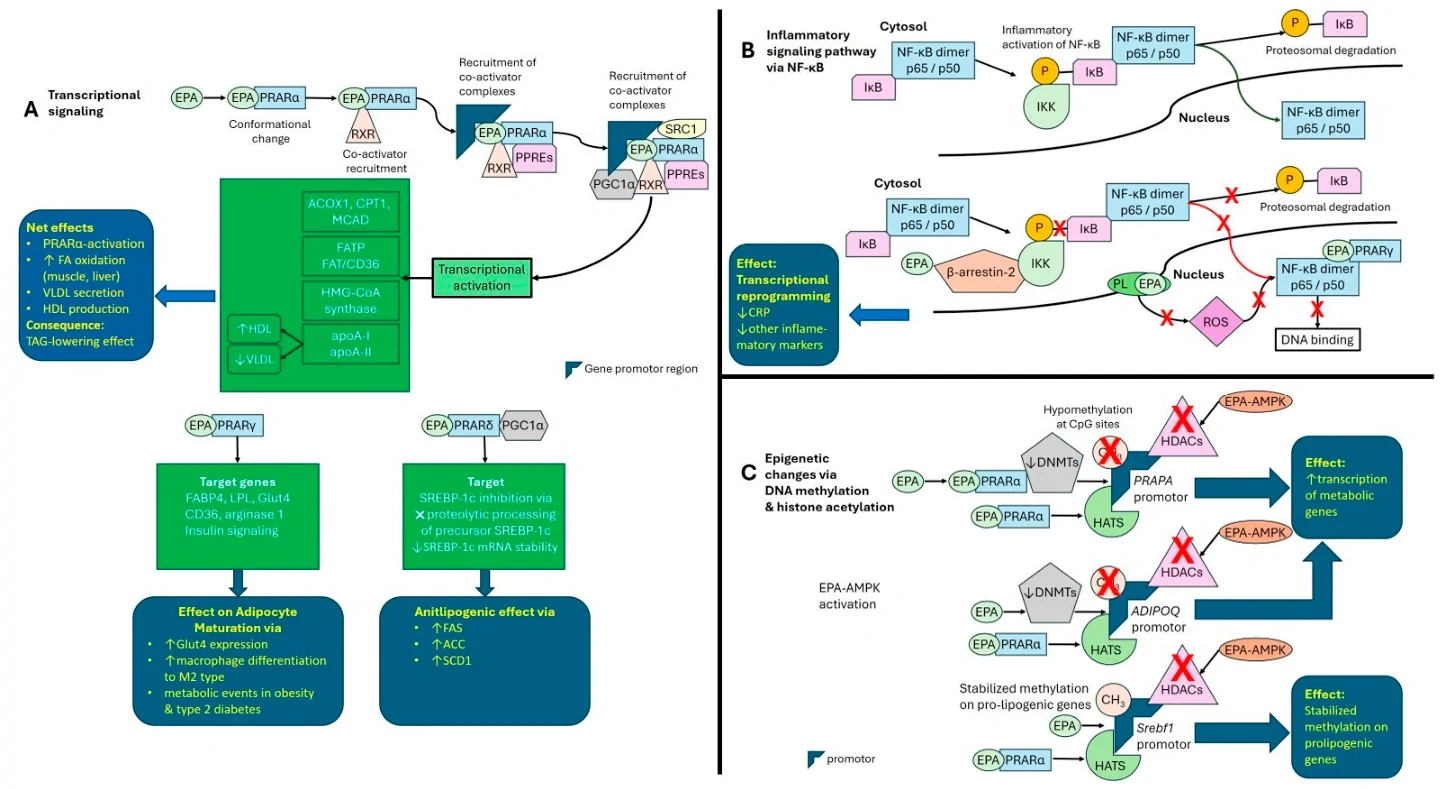
Principal effects of dietary EPA on gene expression, signaling pathways, and epigenetic modifications. (**A**) Transcriptional signaling. EPA binds to PRAR, which then forms a heterodimer with RXR. This complex binds to PPREs located in the promotor region of target genes. Then PGC1 binds to the complex functioning to enhance gene transcription. SRC1 has intrinsic HAT function, and its binding relaxes the surrounding chromatin structure, making access to the DNA possible. EPA-PRARγ and EPA-PRARδ-PGC1 act similarly on different target genes. AAC, acetyl-CoA carboxylase; ACOX, acyl-CoA oxidase; apoA, apolipoprotein A; CD36, cluster of differentiation 36; HMG-CoA synthase, 3-hydroxy-3-methylglutaryl-CoA synthase; CPT, carnitine palmitoyl-transferase; FABPL, liver-type acid binding protein; FAS, fatty acid synthase; FATP, fatty acid transport protein; HDL, high-density lipoprotein; LPL, lipoprotein lipase; Glut4, glucose transporter type 4; MCAD, medium-chain acyl-CoA dehydrogenase; PGC, peroxisome proliferator-activated receptor gamma co-activator, PPREs, peroxisome proliferator response elements; PRAR, peroxisome-proliferating-activated receptor; RXR, retinol X receptor; SCD1, steryl-CoA desaturase 1; SRC, steroid receptor coactivator; SREBP, sterol regulatory element-binding protein; VLDL, very-low-density lipoprotein. (**B**) Inflammatory signaling pathway via NF-κB. Top: Inflammatory signaling pathway. Bottom: EPA effects on the inflammatory pathway. EPA binds to β-arrestin 2, which then binds to IKK, preventing the phosphorylation of IκB and its subsequent degradation. This keeps NF-κB inactive in the cytosol and disallows nuclear translocation. IκB, inhibitor of κB; IKK, IκB kinase complex; NF-κB, nuclear factor kappa light-chain-enhancer of activated B cells; PL-EPA, phospholipid eicosapentaenoic acid; ROS, reactive oxygen species. (**C**) Epigenetic changes via DNA methylation and histone acetylation. EPA binds to and activates PRARα, which reduces the expression of DNMTs, leading to hypomethylation of the *PRARA* promotor. This yields an open chromatin structure, allowing the transcriptional machinery to access the DNA. The effect is further enhanced by the downregulation of HDACs by EPA and the recruitment of HATs in response to EPA-PRARα. The processes are similar for activity of adiponectin and SREBP-1c. *ADIPOQ,* adiponectin gene; DNMTs, DNA methyltransferase; EPA-AMPK, eicosapentaenoic acid-influenced adenosine monop1hosphate-activated protein kinase pathway; HATs, histone acetyl transferases; HDACs, histone deacetylases; *Srebf1,* sterol regulatory element-binding protein 1 gene.

The genomic and signaling effects of EPA exhibit pronounced cell type and context dependency. The molecular mechanisms by which EPA modulates cellular function converge on gene regulatory networks and integrated signaling pathways that reprogram cellular phenotypes. As described previously, EPA influences gene expression through direct transcriptional mechanisms (ligand-activated nuclear receptors), indirect signaling pathway modulation (kinase cascades and transcription factor activation), and epigenetic modifications (DNA methylation and histone modifications) [102]. These genomic effects translate acute biochemical changes into sustained alterations in cellular metabolism, inflammatory responsiveness, and stress resistance, Figure 5 [71].

In transcriptional regulation via nuclear receptors, EPA and EPA-CoA function as endogenous PPAR ligands, binding to the ligand-binding domains of peroxisome proliferator-activated receptors—PPARα, PPARγ, and PPARδ (PPARβ/δ)—and inducing conformational changes that promote coactivator recruitment and transcriptional activation [71,72], Figure 5. PPARα, predominantly expressed in the liver, heart, kidney, and skeletal muscle, is activated by EPA at physiologically relevant concentrations (EC_50_ ~10–50 μM for EPA) [103]. Upon ligand binding, the PPARα–retinoid X receptor (RXR) heterodimer binds peroxisome proliferator response elements (PPREs) in promoter regions of target genes, recruiting coactivator complexes (PGC-1α and SRC-1) and activating transcription [104]. PPARα target genes include the following: 1. FA oxidation enzymes: acyl-CoA oxidase (ACOX1), carnitine palmitoyltransferase 1 (CPT1), and medium-chain acyl-CoA dehydrogenase (MCAD), increasing mitochondrial and peroxisomal β-oxidation capacity (see Supplementary Information); 2. fatty acid transport proteins: FATP and FAT/CD36, enhancing cellular fatty acid uptake [104]; 3. ketogenic enzymes: HMG-CoA synthase, promoting ketone body production during fasting [104]; and 4. lipoprotein metabolism genes: apoA-I, apoA-II (increasing HDL synthesis), and lipoprotein lipase (enhancing VLDL clearance). The net effect of PPARα activation by EPA is increased hepatic and muscle FA oxidation, reduced hepatic VLDL secretion, and increased HDL production, mechanisms underlying the TAG-lowering effects [71]. Clinical studies demonstrate that EPA supplementation (2–4 g/day) reduces plasma TAGs by 20–30%, correlating with upregulation of PPARα target genes in circulating leukocytes and the liver (assessed via biopsy or indirect biomarkers) (see Supplementary Information). PPARγ, expressed primarily in adipose tissue, macrophages, and the colon, is more modestly activated by EPA (EC_50_ ~50–100 μM) compared to synthetic thiazolidinedione agonists. PPARγ target genes include adipocyte differentiation markers (FABP4, LPL, and GLUT4), anti-inflammatory genes in macrophages (CD36 and arginase-1), and insulin signaling components [105]. EPA-mediated PPARγ activation promotes adipocyte maturation, enhances insulin sensitivity via increased GLUT4 expression, and polarizes macrophages toward anti-inflammatory M2 phenotypes [106]. These effects contribute to EPA’s beneficial metabolic actions in obesity and type 2 diabetes [69]. In contrast, PPARδ (PPARβ/δ), ubiquitously expressed but particularly abundant in skeletal muscle, is activated by EPA and regulates genes involved in FA oxidation, mitochondrial biogenesis (via PGC-1α upregulation), and exercise adaptation [107]. EPA-mediated PPARδ activation may underlie improvements in exercise performance and metabolic flexibility observed in some intervention studies [107].

In contrast to PPAR activation, EPA suppresses sterol regulatory element-binding Proteins (SREBPs), Figure 5. EPA inhibits SREBP-1c, the master transcriptional regulator of lipogenic enzymes [73], which is activated by insulin and glucose, inducing expression of FA synthase (FAS), acetyl-CoA carboxylase (ACC), and stearoyl-CoA desaturase (SCD1), promoting de novo lipogenesis in liver and adipose tissue [108]. Inhibition is achieved through multiple mechanisms, such as reducing its gene transcription, inhibiting proteolytic processing of precursor SREBP-1c in the endoplasmic reticulum (preventing nuclear translocation of active SREBP-1c), and reducing SREBP-1c mRNA stability [73], resulting in reduced de novo lipogenesis and lower hepatic TAG content [73]. This anti-lipogenic effect complements PPARα-mediated FA oxidation, creating a dual mechanism for reducing hepatic and plasma TAGs [100]. Clinical studies in non-alcoholic fatty liver disease (NAFLD) demonstrate that EPA supplementation reduces hepatic fat content by 10–30%, correlating with decreased SREBP-1c activity and increased PPARα target gene expression [109].

EPA modulates inflammatory signaling pathways via the nuclear factor-κB (NF-κB) pathway, Figure 5. NF-κB is a master transcriptional regulator of inflammatory gene expression, controlling cytokines (TNF-α, IL-1β, and IL-6), chemokines (MCP-1 and IL-8), adhesion molecules (ICAM-1 and VCAM-1), and pro-inflammatory enzymes (PTGS-2 and iNOS) [110]. In resting cells, NF-κB dimers (typically p65/p50) are sequestered in the cytoplasm by inhibitory IκB proteins [110]. Upon inflammatory stimulation (LPS, TNF-α, and oxidative stress), the IκB kinase (IKK) complex phosphorylates IκB, marking it for proteasomal degradation and allowing NF-κB nuclear translocation and target gene activation. EPA and EPA derivates suppress NF-κB signaling at multiple nodes [50] through the following mechanisms: 1. inhibition of IKK particularly by resolvins; Figure 3), preventing phosphorylation and degradation of IκB; 2. binding to FFA4 recruiting β-arrestin-2, which sequesters TAK1 (a kinase upstream of IKK), blocking inflammatory signal transduction [68]; 3. physical interaction of PPARγ with NF-κB subunits, preventing DNA binding and transcriptional activity; and 4. PL-EPA reduces lipid peroxidation-induced ROS that activate NF-κB [19]. Gene expression profiling in EPA-treated macrophages, monocytes, and endothelial cells demonstrated broad suppression of NF-κB target genes, including 50–80% reductions in TNF-α, IL-1β, IL-6, MCP-1, and PTGS-2 mRNA following inflammatory stimulation. This transcriptional reprogramming underpins the systemic anti-inflammatory effects of EPA and contributes to reduce C-reactive protein (CRP) and other inflammatory biomarkers in clinical trials [111].

EPA also targets the mitogen-activated protein kinase (MAPK) pathways: The MAPK superfamily—including ERK1/2, JNK, and p38 MAPK—transduces extracellular stimuli into transcriptional responses via phosphorylation cascades (see Supplementary Information). EPA modulates MAPK signaling in a context-dependent fashion: in inflammatory settings, EPA generally attenuates JNK and p38 activation (reducing AP-1 and ATF2-mediated inflammatory gene transcription), while in certain growth/survival contexts, EPA may transiently activate ERK1/2 (promoting cell survival or differentiation). The mechanisms of MAPK modulation include altered membrane lipid raft composition affecting upstream kinase localization [34], direct effects of EPA metabolites on phosphatases (particularly MAPK phosphatase-1, which dephosphorylates and inactivates MAPKs), and crosstalk with AMPK signaling [112,113]. The net effect in inflammatory cells is reduced MAPK-mediated inflammatory gene expression, complementing NF-κB suppression [50].

In addition, EPA activates AMPK, which functions as a cellular energy sensor, activated by increased AMP:ATP ratios during energetic stress (exercise, nutrient deprivation, and hypoxia) [113]. EPA-mediated AMPK activation phosphorylates numerous metabolic enzymes and transcription factors, promoting catabolic pathways (FA oxidation and glucose uptake) while inhibiting anabolic pathways (FA and protein synthesis) [114], contributing to improved insulin sensitivity, enhanced FA oxidation, reduced hepatic steatosis, and metabolic flexibility [114]. Clinical studies demonstrate that EPA supplementation increases skeletal muscle AMPK phosphorylation and PGC-1α expression, correlating with improved glucose disposal and reduced fasting insulin. EPA activates AMPK through multiple mechanisms: 1. increasing the cellular AMP:ATP ratio via effects on mitochondrial uncoupling or respiratory efficiency; 2. indirect activation through adiponectin receptor signaling, increasing adiponectin secretion from adipocytes [115]; and 3. potential direct effects on upstream kinases (LKB1 and CaMKKβ) [113]. Through phosphorylation, activated AMPK inactivates ACC (acetyl-CoA carboxylase), reducing malonyl-CoA production and SREBP-1c processing, thereby reducing de novo lipogenesis while increasing FA oxidation relieving CPT1 inhibition [116]. In contrast, it activates PGC-1α, promoting mitochondrial biogenesis, oxidative metabolism, and exercise adaptation [77], while inhibiting mTOR, which inhibits mTOR signaling, reducing protein synthesis and promoting autophagy (see Supplementary Information).

EPA also enhances insulin signaling through multiple mechanisms, such as increasing insulin receptor substrate (IRS) tyrosine phosphorylation, reducing serine phosphorylation (which inhibits IRS), activating Akt/PKB (downstream effector of insulin signaling), and increasing GLUT4 translocation to the plasma membrane [117]. These effects are mediated through anti-inflammatory mechanisms (NF-κB and JNK suppress insulin signaling; EPA reverses this) [118], membrane lipid composition changes affecting insulin receptor function, and PPARγ-mediated transcriptional effects [115]. In insulin-resistant states (obesity and type 2 diabetes), chronic inflammation and lipid accumulation impair insulin signaling [119]. EPA supplementation reduces inflammatory mediators, decreases ectopic lipid deposition (liver and muscle), and restores insulin sensitivity, as evidenced by improved HOMA-IR (Homeostatic Model Assessment of Insulin Resistance) and reduced fasting glucose in clinical trials [111].

Beyond acute transcriptional regulation, EPA induces epigenetic changes that confer long-term alterations in gene expression patterns and cellular phenotypes [102], Figure 5. DNA methylation, primarily at CpG dinucleotides in gene promoters, generally represses transcription [120]. EPA influences DNA methylation through effects on methyl donor availability (via one-carbon metabolism) and DNA methyltransferase (DNMT) activity [120]. Studies in animal models demonstrate that maternal EPA supplementation alters offspring DNA methylation patterns in metabolic genes (PPARα, SREBP-1c, and adiponectin), with persistent effects on metabolic phenotype into adulthood. In human studies, EPA supplementation modestly altered methylation of inflammatory gene promoters (TNF-α and IL-6), potentially contributing to sustained anti-inflammatory effects. Histone acetylation (associated with transcriptional activation) and histone methylation (context-dependent activation or repression) are also modulated by EPA [121]. EPA-activated PPARα recruits histone acetyltransferases (HATs) to target gene promoters, increasing histone H3 and H4 acetylation and chromatin accessibility [121], while EPA-mediated NF-κB inhibition reduces recruitment of HATs to these locations [122]. AMPK activation by EPA influences histone deacetylases (HDACs) and sirtuins (NAD^+^-dependent deacetylases), contributing to metabolic reprogramming and longevity pathways [112].

EPA also modulates expression of microRNAs (miRNAs) in multiple cell types. miRNAs are small non-coding RNAs that post-transcriptionally regulate gene expression by binding target mRNAs, leading to degradation or translational repression [123]. In endothelial cells, EPA increases miR-126 (pro-angiogenic and anti-inflammatory) and decreases miR-155 (pro-inflammatory). In hepatocytes, EPA reduces miR-122 (regulating lipid metabolism) and alters miR-33 (regulating cholesterol homeostasis and FA oxidation) (see Supplementary Information). These miRNA changes contribute to coordinated regulation of metabolic and inflammatory gene networks, amplifying EPA’s transcriptional effects [123].

The concerted cellular effects of EPA are dose- and exposure-type-dependent, affecting genomic and signaling differently based on cell type and tissue (Table 3). In macrophages, dominant effects include NF-κB suppression, PPARγ activation, and M2 polarization (anti-inflammatory phenotype) [106]. In hepatocytes, PPARα activation and SREBP-1c suppression predominate, driving FA oxidation and reduced lipogenesis. In adipocytes, EPA promotes differentiation, adiponectin secretion, and insulin sensitization via PPARγ [105]. In endothelial cells, EPA enhances nitric oxide production, reduces adhesion molecule expression, and promotes anti-thrombotic phenotypes [11]. Temporal dynamics creates short-term (hours) exposure, primarily affecting signaling pathways (AMPK, MAPK, and NF-κB) [76], triggering adaptive responses (e.g., reduced acute inflammatory cytokine release), while long-term exposure (days to weeks) induces transcriptional reprogramming (PPAR target genes and SREBP target genes) and epigenetic changes that stabilize altered phenotypes [71], enabling metabolic remodeling (e.g., increased oxidative capacity and reduced adiposity) [71]. Dose dependency introduces additional complexity: low EPA concentrations preferentially engage high-affinity pathways (FFA4 receptor and certain PPAR interactions) [115], while higher concentrations saturate these pathways and recruit additional mechanisms (membrane biophysical effects and competitive ARA displacement) [71]. Clinical translation requires matching tissue EPA levels (determined by dose and pharmacokinetics) to threshold concentrations for pathway engagement [124].

Integration of mechanisms across pathways identifies non-linear, synergistic effects. For example, AMPK activation phosphorylates PGC-1α, which co-activates PPARα, which transcribes FA oxidation genes, a three-tier amplification cascade [77]. Similarly, NF-κB suppression via IKK inhibition is reinforced by PPARγ trans-repression and FFA4-mediated TAK1 sequestration [125]. This multi-pathway convergence explains why the effects of EPA exceed predictions from single-pathway modeling and underscores the importance of systems-level analysis [126].

### 7.1. Health Effects of EPA and DHA

The biosynthetic sequence ALA → EPA → DHA creates metabolic dependencies. EPA cannot be synthesized without ALA as the precursor, and DHA synthesis requires EPA as an intermediate (in organisms with active Δ4-DES) [127]. However, dietary intake of preformed EPA (from marine sources or *N. oceanica*) bypasses the inefficient ALA → EPA conversion, while dietary DHA bypasses the entire pathway [4]. This metabolic logic explains why direct consumption of long-chain ω-3 EFAs (EPA and DHA) is nutritionally superior to reliance on ALA conversion. The molecular mechanisms, pharmacokinetic properties, and genomic effects of EPA converge to produce clinically significant health benefits across multiple disease domains, such as cardiovascular, inflammation and immune function, metabolic health, and neurological and psychiatric effects (Table 4). Long-term safety data extending 5–10 years from major trials (REDUCE-IT, JELIS, and VITAL) demonstrate sustained benefits without emerging safety signals [5,6,128]. Unlike pharmaceutical interventions that may exhibit tachyphylaxis (diminishing efficacy over time), EPA’s effects on ARA:EPA ratio, inflammatory markers, and clinical outcomes persist with continued use, reflecting sustained incorporation into tissue PLs and stable metabolic reprogramming (Section 5 and Section 6) [100].

Cardiovascular health benefits (Table 4) are the most robustly established clinical effect of EPA through dose-dependent reduction of plasma TAGs [95]. Clinical trials demonstrate 20–30% TAG reduction at 2–4 g/day EPA, with FDA-approved prescription formulations (icosapent ethyl, 4 g/day pure EPA ethyl ester) achieving 25–30% reductions in patients with hypertriglyceridemia [5,93]. The REDUCE-IT trial (Reduction of Cardiovascular Events with Icosapent Ethyl–Intervention Trial), a landmark randomized controlled trial of 8179 patients with elevated TAG levels and cardiovascular disease or diabetes, demonstrated that 4 g/day icosapent ethyl reduced major adverse cardiovascular events (cardiovascular death, myocardial infarction, stroke, coronary revascularization, and unstable angina) by 25% over 4.9 years compared to the placebo [5]. Mechanistically, benefits were attributed to TAG-lowering, anti-inflammatory effects (reduced hsCRP), membrane stabilization, and plaque stabilization. Notably, achieved median EPA levels were ~150 μg/mL (versus ~25 μg/mL placebo), corresponding to ARA:EPA ratios of ~1:1 to 2:1 (optimal range) [129]. Anti-arrhythmic effects of EPA are achieved through incorporation into cardiomyocyte membranes, modulating cardiac ion channels [37], such as changing the inactivation kinetics of the sodium channel (Nav1.5), reducing late sodium current, and preventing calcium overload that triggers arrhythmias [38] (Table 4). L-type calcium channels and potassium channels are similarly modulated, prolonging effective refractory period and stabilizing electrical activity [38]. The JELIS trial (Japan EPA Lipid Intervention Study) in 18,645 patients demonstrated a 19% reduction in major coronary events with 1.8 g/day EPA plus statin versus statin alone, with subgroup analyses suggesting particular benefit in patients with prior arrhythmias [6]. Improved endothelial function is achieved via reduced monocyte adhesion to endothelium, limiting early atherosclerotic lesion formation. These outcomes are the result of increased nitric oxide (NO) production via Akt-mediated eNOS phosphorylation, reducing oxidative stress (decreased NADPH oxidase; increased antioxidant enzymes via Nrf2 activation), and decreased expression of endothelial adhesion molecules (VCAM-1, ICAM-1, and E-selectin) via NF-κB suppression. Imaging studies in established atherosclerosis demonstrate that EPA supplementation (1.8–4 g/day for 6–24 months) reduces plaque volume, increases fibrous cap thickness, and decreases lipid core size in coronary and carotid arteries. EPA stabilizes plaques through the following mechanisms: 1. reduced macrophage accumulation and foam cell formation; 2. decreased matrix metalloproteinase expression (reducing cap thinning); 3. increased collagen synthesis (strengthening fibrous cap); and 4. enhanced efferocytosis (Figure 3, Table 2), clearing apoptotic cells that would otherwise form necrotic cores. Meta-analyses of randomized controlled trials demonstrate that EPA supplementation results in modest but consistent blood pressure reductions (~ 2–4 mmHg systolic and 1–3 mmHg diastolic at doses ≥2 g/day) [99] (Table 4). Mechanisms include increased prostacyclin I_3_ (vasodilatory; Figure 3) [48], enhanced NO bioavailability, reduced endothelin-1 (vasoconstrictor), and improved arterial compliance via membrane effects on vascular smooth muscle cells.

In chronic inflammatory diseases (Table 4), therapeutic benefits result from the anti-inflammatory and pro-resolving properties of EPA (Figure 3, Table 2) [11,59]. Meta analysis of EPA effects on rheumatoid arthritis show that EPA supplementation (2–3 g/day) reduces joint pain, morning stiffness, and tender joint count by 20–30%, with parallel reductions in inflammatory markers (CRP, IL-6, and TNF-α) [130]. Mechanisms include reduced production of pro-inflammatory prostaglandins and leukotrienes from synovial cells and infiltrating immune cells, increased resolvin production promoting inflammation resolution [67], and suppression of NF-κB-mediated inflammatory gene expression in synoviocytes and macrophages. In inflammatory bowel disease (Crohn’s disease and ulcerative colitis), EPA exhibits mixed efficacy: some trials demonstrate maintenance of remission and reduced corticosteroid requirements, while others show minimal benefit. Mechanistic studies reveal that EPA reduces intestinal epithelial NF-κB activation, decreases neutrophil infiltration (via reduced LTB_4_ and increased LTB_5_; Table 2), and enhances intestinal barrier function [131]. Variable clinical efficacy may reflect disease heterogeneity, formulation differences (enteric vs. non-enteric coating), and baseline ω-3 FA status.

EPA modulates both innate and adaptive immunity (Table 4). In innate immunity, EPA reduces TLR4 signaling in macrophages and dendritic cells, decreasing pro-inflammatory cytokine production while enhancing phagocytic capacity and antimicrobial functions [36]. This creates a “resolving” rather than “suppressed” immune phenotype: immune cells remain functional but produce less collateral tissue damage [132]. In adaptive immunity, EPA influences T-cell differentiation, promoting regulatory T cells (Tregs) over pro-inflammatory Th1 and Th17 subsets, partially through effects on antigen-presenting cell function and cytokine milieu. Beyond rheumatoid arthritis, EPA shows promise in other autoimmune diseases. In systemic lupus erythematosus, small trials demonstrate reduced disease activity scores and inflammatory markers with EPA supplementation [130]. In psoriasis, topical and systemic EPA reduce plaque severity, erythema, and scaling, correlating with decreased epidermal ARA content and reduced LTB_4_ production (Figure 3, Table 2) [133]. Larger, definitive trials are needed to establish clinical efficacy and optimal dosing.

EPA supplementation has beneficial effects on metabolic health (Table 4). Clinical trials in type 2 diabetes demonstrate that EPA supplementation (1–4 g/day) reduces fasting glucose by 5–10 mg/dL, improves HOMA-IR by 10–20%, and modestly reduces HbA1c (0.2–0.4%), with greater effects in patients with baseline hypertriglyceridemia or elevated inflammatory markers [111,134]. Metabolic health benefits are attributed to improved insulin sensitivity through anti-inflammatory mechanisms (reducing TNF-α and IL-6 that impair insulin signaling) [118], enhanced AMPK activation promoting glucose uptake, increased adiponectin secretion from adipocytes [115], and reduced ectopic lipid deposition in muscle and liver. Non-alcoholic fatty liver disease (NAFLD) (Table 4) affects 25–30% of adults in developed countries and is strongly associated with insulin resistance and cardiovascular risk [135]. Clinical trials using MRI or ultrasound demonstrate that EPA supplementation (1.8–2.7 g/day for 6–12 months) reduces hepatic fat content by 10–30%, with improvements in liver enzymes (ALT and AST) and inflammatory markers [109]. Some trials show reduced liver fibrosis scores, though longer-term studies are needed to confirm anti-fibrotic efficacy. Reduction in lipid contents are achieved through the following mechanisms: 1. PPARα-mediated FA oxidation [71]; 2. reduced de novo lipogenesis through suppression of SREBP-1c [73]; 3. AMPK activation [114]; and 4. anti-inflammatory effects reducing hepatocyte stress and fibrosis progression [136]. In contrast, fighting obesity with EPA seems to be less promising, as consistent significant weight loss in clinical trials (typical reductions <1–2 kg) was not achieved. Nonetheless, in adipose tissue, EPA was shown to promote adipocyte differentiation and “metabolically healthy” adipose expansion (subcutaneous over visceral) [2], increasing adiponectin secretion (insulin-sensitizing adipokine), reducing macrophage infiltration and inflammatory cytokine production, and improving insulin sensitivity. These effects dissociate obesity from metabolic dysfunction, potentially explaining why EPA improves metabolic parameters even without major weight reduction. Metabolic syndrome—clustering of abdominal obesity, hypertriglyceridemia, low HDL, hypertension, and impaired fasting glucose—affects ~35% of U.S. adults and confers elevated cardiovascular and diabetes risk [137]. EPA addresses multiple syndrome components simultaneously (Table 4): reducing TAGs (20–30%) [95], modestly raising HDL (5–10%) [111], lowering blood pressure (2–4 mmHg systolic) [99], improving glucose homeostasis (5–10 mg/dL fasting glucose reduction) [111], and reducing visceral adiposity and inflammatory markers [5,129]. Meta-analyses suggest EPA supplementation (≥2 g/day) can reverse metabolic syndrome classification in 15–25% of patients over 6–12 months, with reductions in the ARA:EPA ratio serving as a mechanistic biomarker of multi-component improvement (Table 5) [100].

EPA supplantation may be effective in managing major depressive and mood disorders (Table 4), improve cognitive function, and offer neuroprotection [90,138]. Meta-analyses of randomized controlled trials with ≥1 g/day pure EPA or EPA-dominant formulation show significant anti-depressant effects (standardized mean difference ~0.4–0.5 versus placebo), comparable to effect sizes of conventional anti-depressants in mild-to-moderate depression [139]. Mechanistic hypotheses include anti-inflammatory effects (depression is associated with elevated IL-6, TNF-α, and CRP), enhanced membrane fluidity affecting neurotransmitter receptor function, increased brain-derived neurotrophic factor (BDNF) expression, and modulation of HPA axis stress responses. Despite higher concentrations of DHA in the brain, EPA appears more effective (see Supplementary Information). This paradox may be due to the superior anti-inflammatory properties of EPA (Figure 3, Table 2) and anti-inflammatory mechanisms (reducing cytokine signaling-induced inflammation) rather than direct brain incorporation. An erythrocyte ARA:EPA ratio of <3:1 could enhance the anti-depressant response (Table 5). While effects of EPA on cognitive function are more modest and more variable than for DHA, consistent with limited brain EPA accumulation, EPA may provide neuroprotection through reducing systemic and neuroinflammation (microglia in brain produce inflammatory mediators, ARA; EPA competition reduces this; Figure 3) [140], improving cerebrovascular function (enhanced endothelial function and reduced microvascular inflammation), and potential indirect effects via increased DHA synthesis from EPA-derived DPA, though conversion efficiency may be too low. In neurodegenerative diseases (e.g., Alzheimer’s and Parkinson’s diseases), EPA shows promise in preclinical models, but clinical trial results are inconsistent. For cognitive improvement, combined EPA + DHA formulations appear to be more effective than EPA alone, suggesting complementary mechanisms, with EPA possibly providing primarily anti-inflammatory and vascular protection, while DHA provides direct neuronal membrane support. The limited blood–brain barrier transport of EPA relative to DHA constrains direct central nervous system effects [90]. For psychiatric conditions where peripheral inflammatory mechanisms dominate (depression and anxiety), EPA is highly effective [138]. For conditions requiring direct neuronal membrane modification (cognitive decline and neurodevelopmental disorders), DHA or EPA + DHA combinations are preferred. Understanding this mechanistic distinction guides rational ω-3 EFA selection for neuropsychiatric applications (see Supplementary Information).

Other clinical applications investigating eye, pregnancy, and maternal health and benefits in the treatment of cancer use mixtures of EPA and DHA. EPA’s role in retinal function is limited compared to DHA (the predominant fatty acid in retinal photoreceptor membranes) [141]. However, EPA contributes to retinal vascular health through anti-inflammatory and anti-angiogenic effects relevant to age-related macular degeneration (AMD) and diabetic retinopathy [142]. The AREDS2 trial (Age-Related Eye Disease Study 2) tested combined EPA + DHA supplementation in AMD, showing modest benefits in reducing progression to advanced AMD, though discerning EPA-specific effects is difficult due to the co-administration of DHA [143]. During pregnancy, ω-3 EFAs, particularly DHA, are critical for fetal neurodevelopment [144], while the role of EPA is primarily supportive, reducing maternal inflammation, improving placental function, and potentially reducing pre-eclampsia risk through anti-inflammatory and vasodilatory effects [144]. Recommendations for pregnancy emphasize combined EPA + DHA (with DHA ≥200 mg/day for fetal brain development) supplementation for healthy development of the fetus [144]. Preclinical studies demonstrate anti-cancer effects of EPA in various cancers through inhibiting cancer cell proliferation, inducing apoptosis, suppressing angiogenesis, and reducing metastasis. Mechanisms include altered membrane PL composition disrupting growth factor receptor signaling [145], increased oxidative stress (paradoxical pro-oxidant effect at high concentrations), PTGS-2 inhibition reducing pro-tumorigenic prostaglandins [145], and immune modulation enhancing anti-tumor immunity [145]. However, clinical trial evidence is limited and mixed, with most studies using combined EPA + DHA [145]. EPA may have supportive roles in cancer cachexia (preserving lean body mass) and reducing chemotherapy side effects, but definitive evidence for anti-cancer efficacy in humans is currently lacking.

### 7.2. ARA:EPA Ratio as a Clinical Biomarker and Therapeutic Target

The ARA:EPA ratio in plasma PL or erythrocyte membranes integrates dietary intake, minimal endogenous biosynthesis of EPA, competitive incorporation dynamics (Figure 3), and tissue-level metabolic consequences into a single quantitative biomarker (Table 5) [8,79]. This ratio serves dual functions: risk stratification and therapeutic monitoring. Prospective cohort studies demonstrate that individuals with ARA:EPA ratios >10:1 have 2–3-fold elevated risk of cardiovascular events compared to those with ratios <3:1, independent of traditional risk factors (LDL cholesterol, blood pressure, and smoking) (see Supplementary Information). The ratio predicts inflammatory disease activity, metabolic syndrome prevalence, and depression severity across populations [80]. Population reference ranges from Western countries typically show median ratios of 10–15:1 (reflecting low fish intake and high ω-6 vegetable oil consumption), while Japanese and Mediterranean populations with high fish consumption exhibit ratios of 2–5:1, correlating with lower cardiovascular and inflammatory disease rates (Table 5).

**Table 5:** ARA:EPA ratio ranges and associated health profiles.

ARA:EPA Ratio	Category	Dietary Pattern	Inflammatory Status	Disease Risk	Clinical Recommendations
**>15:1**	Very High	Low omega-3, high omega-6	Highly pro-inflammatory	Very high CVD, metabolic	Urgent dietary intervention
**10–15:1**	High	Typical Western	Pro-inflammatory	Elevated CVD, metabolic	EPA supplementation advised
**5–10:1**	Moderate–High	Low fish intake or supplementation	Moderately inflammatory	Moderate CVD risk	Increase omega-3 intake
**3–5:1**	Moderate	Occasional fish or supplementation	Balanced-inflammatory	Moderate risk	Continue current intake
**1–3:1**	Low	Regular fish or supplementation	Anti-inflammatory	Low risk	Optimal maintenance
**<1:1**	Very Low	High EPA supplementation	Highly anti-inflammatory	Very low risk	Excellent status

CVD = cardiovascular disease; CRP = C-reactive protein; IL-6 = interleukin-6.

ARA:EPA ratio testing is commercially available through specialized lipid panels (plasma PL fatty acid analysis or erythrocyte membrane FA analysis) [79]. Costs have decreased with automated gas chromatography and mass spectrometry platforms, making routine measurement feasible in clinical practice [98]. Integration of the ARA:EPA ratio into cardiovascular risk calculators and metabolic syndrome diagnostic criteria is under investigation, potentially elevating this biomarker from a research tool to standard clinical assessment [79,100]. The ratio of ARA to EPA in plasma PL or erythrocyte membranes is an established biomarker of ω-3 FA status, inflammatory balance, and cardiovascular risk (Table 3). Higher ARA:EPA ratios (>5:1) indicate dominance of pro-inflammatory eicosanoid precursors and are associated with elevated inflammatory markers, increased cardiovascular disease risk, and metabolic dysfunction. Moderate ratios (3–5:1) represent transitional status often targeted in initial dietary interventions. Lower ratios (<3:1) achieved through regular fish consumption or EPA supplementation correlate with anti-inflammatory eicosanoid profiles, reduced inflammatory biomarkers (CRP, IL-6), and decreased cardiovascular event rates. Optimal ratios (<1:1) yield EPA-PL integration, and EPA-derived eicosanoids and resolvins quantitatively and qualitatively dominate ARA-derived mediators. In clinical applications, serial ARA:EPA ratio measurement tracks dietary intervention effectiveness, ω-3 FA supplementation compliance and efficacy, and treatment response in inflammatory conditions (rheumatoid arthritis and inflammatory bowel disease). It needs to be taken into account though that individuals exhibit variability in response to EPA supplementation (10-fold range in plasma EPA increases for identical doses) due to genetic polymorphisms in absorption, metabolism, and incorporation pathways. Monitoring the ratio, however, enables dose titration to achieve personalized therapeutic targets [95]. For example, patients with APOE4 genotype (associated with reduced ω-3 FA incorporation) may require higher EPA doses to achieve target ratios compared to non-carriers. Differences in EPA uptake affect clinical outcomes. In depression trials, patients achieving ARA:EPA <3:1 show 60–70% response rates versus 20–30% in those remaining >5:1, enabling stratified treatment approaches (see Supplementary Information). Clinical guidelines regarding therapeutic targets increasingly recognize ARA:EPA ratio targets for disease management (Table 3). Disease-specific targets guide personalized recommendations: general health maintenance (<5:1), cardiovascular disease prevention (<3:1), inflammatory disease management (<2:1), and optimal anti-inflammatory status (<1:1, achievable with high-dose EPA supplementation from sources including *N. oceanica* oils).

## 8. Future Directions

Despite substantial progress, critical knowledge gaps persist across molecular, cellular, and clinical domains, limiting the optimization of EPA-based interventions and mechanistic understanding. Specialized pro-resolving mediator receptor pharmacology remains incompletely characterized. In addition, tissue-specific mechanism profiles exhibit pronounced heterogeneity inadequately captured by current models. EPA’s dominant mechanisms differ fundamentally across cell types, yet the molecular determinants of this context-dependency (differential receptor expression, cofactor availability, competing metabolic pathways, and epigenetic states) remain poorly defined. Comprehensive tissue-resolved lipidomics, transcriptomics, and proteomics following EPA exposure would reveal cell-type-specific vulnerabilities and therapeutic opportunities.

Genetic variation effects on EPA biology represent a largely unexplored frontier despite obvious clinical relevance. Genome-wide association studies (GWAS) correlating genetic variants with plasma/erythrocyte EPA levels, ARA:EPA ratios, and clinical responses to EPA supplementation could enable pharmacogenomic stratification, identifying high responders versus low responders and guiding personalized dosing. Precision medicine applications require integrating multi-omic data (genomics, lipidomics, metabolomics, and microbiomics) with clinical phenotypes to define EPA-responsive patient subgroups. Optimal dosing strategies remain empirically derived rather than mechanistically guided. While clinical trials establish efficacy ranges (1–4 g/day), precision dosing algorithms incorporating individual factors—baseline ARA:EPA ratio, FADS genotype, body composition, inflammatory status, and concurrent medications—do not exist. Developing PK/PD models predicting target tissue EPA concentrations and ARA:EPA ratios from patient-specific parameters would enable dose-individualization maximizing efficacy while minimizing cost and pill burden. Biomarker development beyond the ARA:EPA ratio could enhance therapeutic monitoring and mechanistic insight. Candidate biomarkers include plasma resolvin E levels, eicosanoid metabolite panels, tissue-specific membrane EPA incorporation kinetics, and transcriptional signatures. Validating these biomarkers against clinical outcomes would refine mechanistic understanding and enable real-time therapeutic optimization. Prospective trials stratifying patients by predictive biomarkers could demonstrate precision EPA medicine superiority over empiric supplementation.

Several nascent research areas promise transformative insights into EPA biology and therapeutic applications. Microbiome interactions represent a bidirectional frontier. Recent metagenomic studies suggest that individual microbiome profiles may explain up to 40% of inter-individual variability in EPA bioavailability and tissue incorporation, highlighting the potential for microbiome-guided personalization (see Supplementary Information). Gut microbiota metabolize EPA to bioactive derivatives (hydroxylated-, conjugated FAs) with distinct biological properties; conversely, EPA modulates microbiome composition, favoring anti-inflammatory bacterial taxa (e.g., *Akkermansia muciniphila* and *Faecalibacterium prausnitzii*) while suppressing pro-inflammatory species (see Supplementary Information). Whether microbiome-dependent EPA metabolism contributes to clinical efficacy, whether microbiome composition predicts EPA responsiveness, and whether synbiotic formulations (EPA plus specific probiotics) enhance outcomes warrant investigation. Germ-free animal models colonized with defined microbial communities could dissect microbiome contributions to EPA pharmacology. Nanoparticle encapsulation, self-emulsifying delivery systems, and targeted release formulations could enhance bioavailability, improve tissue targeting, or achieve controlled release kinetics (see Supplementary Information). Recent development of EPA-loaded polymeric nanoparticles achieving 3-fold higher brain bioavailability in preclinical models suggests promise for neurological applications previously limited by blood–brain barrier impermeability.

Combination therapies exploiting mechanistic synergies represent rational polypharmacy. EPA plus AMPK activators (metformin) may synergistically improve insulin sensitivity and metabolic health. EPA plus mitochondrial antioxidants (e.g., CoQ10, MitoQ, and alpha-lipoic acid) could enhance energetic benefits while reducing oxidative stress. EPA plus specialized probiotics selected for anti-inflammatory properties may optimize microbiome-mediated benefits. Systematic screening of EPA combinations in disease-relevant models followed by biomarker-driven clinical trials could identify high-value pairings for challenging conditions like treatment-resistant depression or advanced NAFLD.

## 9. Conclusions

This review has constructed a comprehensive molecular-to-clinical framework demonstrating how the specific positioning of the Δ17 double bond of EPA governs competitive enzymatic metabolism with ARA. Central to EPA’s therapeutic efficacy is its capacity to shift the ARA:EPA ratio, a unifying biomarker that encapsulates membrane dynamics, inflammatory balance, and metabolic health, thereby offering a quantifiable target for intervention across a broad spectrum of chronic diseases. Precision medicine approaches identifying EPA-responsive patient subgroups, optimizing dosing algorithms, and combining EPA with complementary therapies could expand therapeutic utility across the disease spectrum. These insights position EPA as a systems-level regulator of cellular function, linking molecular structure to clinical outcome through tightly integrated biochemical and transcriptional networks, yet translating mechanistic knowledge into actionable clinical tools that deliver EPA’s promise of sustainable, safe, and effective disease prevention and treatment to the diverse global population will require multi-omics technologies, systems biology modeling, precision medicine frameworks, and sustainable EPA-dominant oil production, for which *N. oceanica* is a prime candidate.

## Figures and Tables

**Table 1:** Characterized fatty acid desaturases and elongases in *Nannochloropsis oceanica* CCMP531/1779.

Enzyme	GenBank ID	Length (aa)	EC Number	Function	Expression
**Δ9-DES**	AQX92133.1	359	1.14.19.1	18:0 → 18:1Δ9	Constitutive
**Δ12-DES**	AQX92134.1	438	1.14.19.6	18:1 → 18:2Δ9,12	High
**Δ6-DES**	AQX92135.1	474	1.14.19.3	18:3 → 18:4Δ6	High
**Δ6-ELOVL**	AQX92136.1	276	2.3.1.199	18:4 → 20:4	High
**Δ5-DES**	AQX92137.1	526	1.14.19.37	20:4 → 20:5 (EPA)	High
**Om**	AQX92138.1	410	Unknown	Unknown	Unknown
**C20→C22 ELOVL**	Not char.	-	2.3.1.199	20:5 → 22:5	Low/absent
**Δ4-DES**	Not char.	-	1.14.19.44	22:5 → 22:6 (DHA)	Low/absent

(aa = amino acids; char. = characterized; DES = desaturase; ELOVL = elongase).

**Table 2:** Comparative biological potencies of ARA- vs. EPA-derived eicosanoids.

Metabolite Pair	ARA Product	EPA Product	Relative Potency	Functional Difference
**Prostaglandins**	PGE_2_	PGE_3_	PGE_2_ >> PGE_3_	PGE_2_ more inflammatory
**Thromboxanes**	TXA_2_	TXA_3_	TXA_2_ >> TXA_3_	TXA_3_ minimal platelet aggregation
**Leukotrienes**	LTB_4_	LTB_5_	LTB_4_ 10–100× > LTB_5_	LTB_5_ weak chemoattractant
**Resolvins**	-	RvE1-3	Unique to EPA	Active resolution signaling

**Table 3:** Tissue, duration, and dosage effects of dietary EPA intake.

Tissue	
Macrophages	Main effect: anti-inflammatory NF-κB suppression PRARγ activation Macrophage polarization M2 anti-inflammatory type
Hepatocytes	Main effect: reduction in TAGs PRAPα activation; SREBP-1c suppression results in ↑ FA oxidation and ↓ lipogenesis
Adipocytes	Main effect: adipocyte differentiation ↑ adiponectin secretion ↑ insulin-sensitization via PRARγ
Endothelial cells	Main effect: enhances nitric oxide production, reduces adhesion molecule expression, and promotes anti-thrombotic phenotypes ↑ NO production ↓ adhesion molecule expression
**Duration**	
Short-term	Main effect: adaptive processes, such as reduced inflammatory cytokine release via effect on AMPK, MAPK, and NF-κB signaling pathways
Long-term	Main effect: transcriptional reprogramming and epigenetic changes via PRAR and SREBP-1c gene targets
**Dosage**	
Low	Main effect: high affinity pathways such as FFA4 receptor and certain PRAR pathways
Moderate to high	Main effect: recruitment of additional pathways that influence membrane biophysical effects through ARA replacement

FA, fatty acid; FFA4, free fatty acid receptor 4; NF-κB, nuclear factor kappa-light-chain-enhancer of activated B cells; NO, nitric oxide; PRARα and -γ, peroxisome proliferator-activated receptor α and -γ, respectively; SREBP-1c, sterol regulatory element-binding-protein-1c; TAG, triacylglycerides.

**Table 4:** Health benefits summary of dietary EPA intake.

Health Target	Health Effect	Proposed Mechanisms
Cardiovascular	Anti-arrhythmic	Incorporation of EPA into cardiomyocytes, leading to modulation of Ca-channels, which changes inactivation of the sodium channel Nav1.5, resulting in reduced sodium currents and prevention of calcium overloads
Blood pressure	↓ Blood pressure	*↑* prostacyclin I^3^ (vasodilator) ↑ nitric oxide availability ↓ endothelin-1 (vasoconstrictor) ↑ arterial compliance via membrane effects on smooth muscle
Chronic inflammation	↓ Inflammation	↓ CRP, IL-6, and TNF-α ↓ inflammatory prostaglandins ↓ leukotrienes ↑ resolving production leading to inflammatory resolution by suppressing NF-κB-mediated inflammatory gene expression
Innate immunity	Creating a resolving phenotype	↓ TLR4 signaling in macrophages and dendritic cells ↓ pro-inflammatory cytokine production ↑ phagocytotic capacity ↑ anti-microbial function
Adaptive immunity	T-cell differentiation	Effects on antigen-presenting cells and cytokine profiles ↑ regulatory T cells (Tregs) ↓ pro-inflammatory Th1 and Th17 T-cell subsets
Metabolic health	Type 2 diabetes	↑ insulin sensitivity through ↓ insulin-impairing inflammatory compounds (TNF-α and IL-6), ↑ AMPK activation, resulting in ↑ glucose uptake, ↑ adiponectin secretion, and ↓ ectopic lipid deposition ↓ fasting glucose Improved HOMA-1R and modest ↓ HbA1c
NAFLD	Reduction in hepatic fat	↑ liver enzymes (ALT, AST) ↓ inflammatory markers ↓ liver fibrosis (in some cases) ↑PRAPα-mediated FA oxidation ↓ de novo lipogenesis through suppression of SREBP-1c and activation of AMPK
Metabolic syndrome	↓ abdominal obesity, ↓ hypertriglyceridemia, ↑ HDL, and ↑ glucose homeostasis	↓ TAGs Modest ↑ HDL ↓ blood pressure ↓ visceral fat ↓inflammatory markers
Mild–moderate depressive mood disorders	Relieves anxiety and depression	↓ inflammatory markers (IL-6, TNF-α, and CRP) ↑ membrane fluidity affecting neurotransmitter function ↑ brain-derived neurotrophic factors (BDNF), leading to modulation of HPA axis stress responses
Neuropsychiatric applications	Constrained by limited blood–brain barrier transport of EPA vs. DHA constrains effectivity to conditions with dominance of peripheral inflammatory mechanisms such as anxiety and mild to moderate depression	See above

ALT, alanine transaminase; AMPK, AMP-activated protein kinase; AST, aspartate transaminase; CRP, c-reactive protein; HbA-1c, glycated hemoglobin; HDL, high-density lipoprotein; HOMA-1R, homeostatic model assessment of insulin resistance; HPA axis, hypothalamic–pituitary–adrenal axis; IL-6, interleukin-6; NAFLD, non-alcoholic fatty liver disease; MAPK, mitogen-activated protein kinase; SREBP-1c, sterol regulatory element-binding-protein-1c; TLR4, toll-like receptor-4; TNF-α, tumor necrosis factor-α; TAGs, triacylglycerides.

## Data Availability

No new data were created or analyzed in this study.

## Supplementary Materials

The following supporting information can be downloaded at: https://www.mdpi.com/article/10.3390/ijms27219834/s1 [15,146,147,148,149,150,151,152,153,154,155,156,157,158,159,160,161,162,163,164,165,166,167,168,169,170,171,172,173,174,175,176,177,178,179,180,181,182,183,184,185,186,187,188,189,190,191,192,193,194,195,196,197,198,199,200,201,202].
