# Supplementary material for: Molecular Science of Eicosapentaenoic Acid (EPA) Omega-3 Fatty Acid Using Nannochloropsis oceanica as a Model Organism
Source: Int J Mol Sci. 2026 Nov 2;27(21):9834. doi: 10.3390/ijms27219834 (PMC13763962; doi:10.3390/ijms27219834)
Supplement: Supplementary file 1 [file ijms-27-09834-s001.zip › ijms-4553408-supplementary.pdf]

Molecular Science of Eicosapentaenoic Acid (EPA) Omega-3 Fatty Acid Using *Nannochloropsis oceanica* as a Model Organism

Stephens, E.D.[1,2,3,*], Thomas-Hall, S.[4], Galea, V.[1] & Heimann and K.[5,*]

1 University of Queensland, Australia
2 Algemy Pty Ltd, Australia and Hong Kong
3 Advantage Plants Consulting Pty Ltd, Australia
4 SeoO2 Pty Ltd, Australia
5 Southern Cross University, Faculty of Science and Engineering, PO Box 157
Military Road East Lismore NSW 2480, Australia
Stephens, E.D., University of Queensland, School of Agriculture and Food Sustainability, St Lucia 4072, Brisbane, Queensland, Australia evan.stephens@uq.edu.au
Heimann, K., Southern Cross University, Faculty of Science and Engineering, PO Box 157, Military Road East Lismore NSW 2480, Australia kirsten.heimann@scu.edu.au

Supplementary Information

S1—Nanno*chloropsis Taxonomy*

*Speci*es:

Historically, seven Nannochloropsis species have been designated, including

N. gaditana;

N. oculata;

N. salina;

N. granulata;

N. limnetica;

N. oceanica;

N. australis.

However, for the genus Nannochloropsis, there are only five species currently recognized:

Nannochloropsis oculata (Droop 1955) Hibberd 1981;

Nannochloropsis granulata Karlson & Potter 1996;

Nannochloropsis limnetica Krienitz et al. 2000;

Nannochloropsis oceanica Suda et al. 2002;

Nannochloropsis australis Fawley et al. 2015.

This is because the species Nannochloropsis gaditana and Nannochloropsis salina were reclassified as Microchloropsis gaditana and Microchloropsis salina, respectively, in 2015 (however, there remain studies since 2015 that have not recognized this reclassification).[15]

Nannochloropsis oceanica CCMP531/CCMP1779

NCBI Taxonomy ID 1027358

https://www.ncbi.nlm.nih.gov/datasets/taxonomy/1027358/[146]

The algae of the genus Nannochloropsis differ from other related microalgae in that they have chlorophyll and completely lack chlorophyll b and chlorophyll c. In addition, they are able to build up high concentrations of a range of pigments such as astaxanthin, zeaxanthin, and *canthaxanthin.*[147, 148]

S2—Biosynthesis pathway links to NIH/NLM/NCBI:

Taxonomy:

Nannochloropsis oceanica CCMP531/CCMP1779
NCBI Taxonomy ID 1027358
https://www.ncbi.nlm.nih.gov/datasets/taxonomy/1027358/

Biosynthesis Pathway:

Delta-9 fatty acid desaturase [Nannochloropsis oceanica]
GenBank: AQX92133.1
https://www.ncbi.nlm.nih.gov/protein/AQX92133.1

Delta-12 fatty acid desaturase [Nannochloropsis oceanica]
GenBank: AQX92134.1
https://www.ncbi.nlm.nih.gov/protein/AQX92134.1

Delta-6 fatty acid desaturase [Nannochloropsis oceanica]
GenBank: AQX92135.1
https://www.ncbi.nlm.nih.gov/protein/AQX92135.1

Delta-6 fatty acid elongase [Nannochloropsis oceanica]
GenBank: AQX92136.1
https://www.ncbi.nlm.nih.gov/protein/AQX92136.1

Delta-5 fatty acid desaturase [Nannochloropsis oceanica]
GenBank: AQX92137.1
https://www.ncbi.nlm.nih.gov/protein/AQX92137.1

Om [Nannochloropsis oceanica]
GenBank: AQX92138.1
https://www.ncbi.nlm.nih.gov/protein/AQX92138.1

S3—Additional Topic-Related Literature

References [146-202] are cited in the supplementary materials
